# A Rab5 endosomal pathway mediates Parkin-dependent mitochondrial clearance

**DOI:** 10.1038/ncomms14050

**Published:** 2017-01-30

**Authors:** Babette C. Hammerling, Rita H. Najor, Melissa Q. Cortez, Sarah E. Shires, Leonardo J. Leon, Eileen R. Gonzalez, Daniela Boassa, Sébastien Phan, Andrea Thor, Rebecca E. Jimenez, Hong Li, Richard N. Kitsis, Gerald W. Dorn II, Junichi Sadoshima, Mark H. Ellisman, Åsa B. Gustafsson

**Affiliations:** 1 Skaggs School of Pharmacy and Pharmaceutical Sciences, University of California San Diego, 9500 Gilman Drive 0758, La Jolla, California 92093, USA; 2 Center for Research in Biological Systems, National Center for Microscopy and Imaging Research, University of California San Diego, La Jolla, California 92093, USA; 3 Rutgers New Jersey Medical School, Newark, New Jersey 07103, USA; 4 Albert Einstein College of Medicine, Bronx, New York 10461, USA; 5 Washington University School of Medicine, St Louis, Missouri 63110, USA

## Abstract

Damaged mitochondria pose a lethal threat to cells that necessitates their prompt removal. The currently recognized mechanism for disposal of mitochondria is autophagy, where damaged organelles are marked for disposal via ubiquitylation by Parkin. Here we report a novel pathway for mitochondrial elimination, in which these organelles undergo Parkin-dependent sequestration into Rab5-positive early endosomes via the ESCRT machinery. Following maturation, these endosomes deliver mitochondria to lysosomes for degradation. Although this endosomal pathway is activated by stressors that also activate mitochondrial autophagy, endosomal-mediated mitochondrial clearance is initiated before autophagy. The autophagy protein Beclin1 regulates activation of Rab5 and endosomal-mediated degradation of mitochondria, suggesting cross-talk between these two pathways. Abrogation of Rab5 function and the endosomal pathway results in the accumulation of stressed mitochondria and increases susceptibility to cell death in embryonic fibroblasts and cardiac myocytes. These data reveal a new mechanism for mitochondrial quality control mediated by Rab5 and early endosomes.

## Abstract (web summary)

Damaged mitochondria are normally cleared through canonical and alternative autophagy pathways. Here, the authors report that mitochondria can be cleared through an autophagy-independent endosomal-lysosomal pathway that depends on Parkin-dependent sequestration of mitochondria in Rab5-positive early endosomes.

Mitochondria are critical for many cellular functions, including energy production, metabolism and cell death. Therefore, maintaining a functional population of mitochondria is critical for the survival of many cells. Mitochondrial dysfunction is implicated in multiple neurodegenerative and cardiovascular diseases. To ensure maintenance of a healthy mitochondrial network, impaired mitochondria are selectively degraded by a process known as autophagy. Conventional autophagy, which requires autophagy-related proteins (Atg), such as Atg5 and Atg7 (refs 1, 2), is well-known to play a role in mitochondrial autophagy or mitophagy^3^. Recent studies have also described the existence of an alternative pathway of autophagy that is independent of Atg5/7 but dependent on Unc-51-like autophagy activating kinase 1 (Ulk1) and Rab9 (ref. 4). There is also evidence that mitochondria can be directly taken up into lysosomes via a process known as microautophagy^5^. Whether additional pathways exist to clear mitochondria is currently unknown.

The E3 ubiquitin ligase Parkin labels mitochondria for autophagic degradation. Loss-of-function mutations in Parkin contribute to loss of dopaminergic neurons in the substantia nigra and cause early on-set of Parkinson's disease^6^. Parkin also plays an important role in clearing damaged mitochondria in the myocardium and in promoting mitophagy of fetal cardiac mitochondria in newborn mice^7^,^8^,^9^,^10^. Parkin is cytosolic under baseline conditions, but rapidly translocates to depolarized mitochondria where it ubiquitinates several different proteins^11^. Interestingly, although ubiquitin can target proteins for degradation via either the autophagic or endocytic pathways, the relationship between Parkin-mediated ubiquitination and the endosomal degradation pathway has not been explored. Despite our growing knowledge of the molecules and pathways involved in mitochondrial clearance, it is still unclear whether autophagy is the only pathway by which cells can eliminate mitochondria.

Here we describe a new mechanism of Parkin-mediated mitochondrial clearance in cells that involves the small GTPase Rab5 and the endosomal degradation pathway. We report that mitochondria are sequestered inside Rab5-positive early endosomes via the endosomal sorting complexes required for transport (ESCRT) machinery and subsequently delivered to lysosomes for degradation. Our data also demonstrate that clearing dysfunctional mitochondria via the endosomal pathway protects against cell death.

## Results

### Parkin directs autophagy-independent mitochondrial clearance

**Figure 1:**
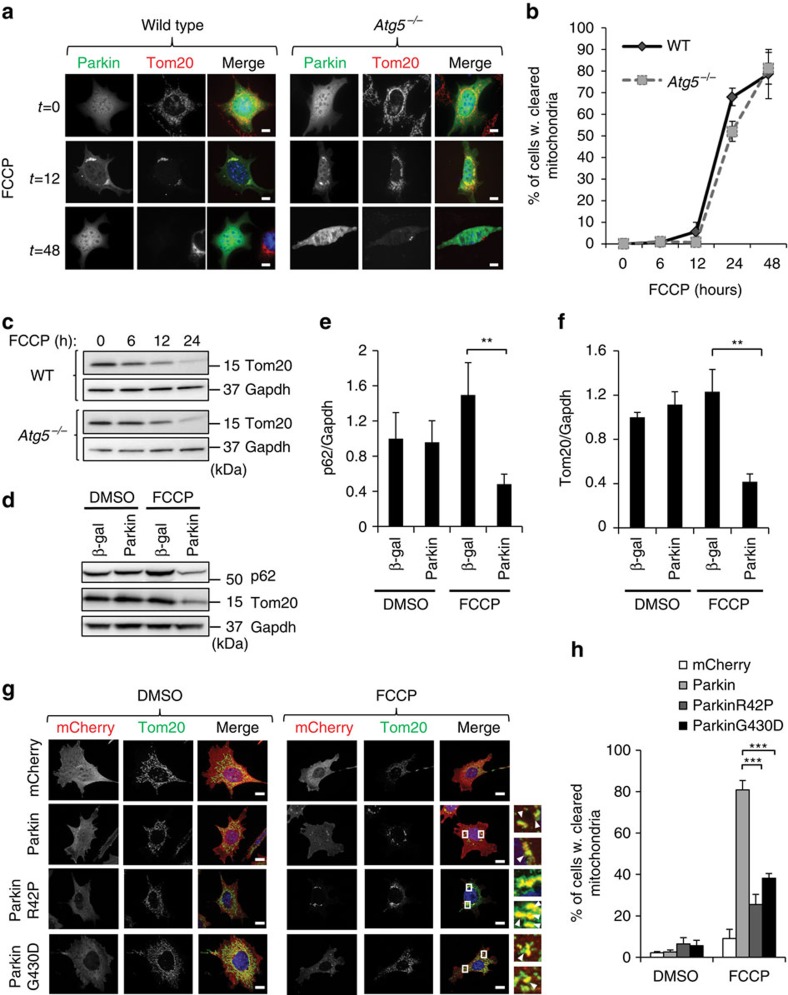
Parkin promotes clearance of mitochondria in autophagy-deficient cells. (**a**) Representative images of WT and *Atg5*^*−/−*^ MEFs overexpressing YFP-Parkin. Cells were fixed at the indicated time points (in hours) after treatment with FCCP (25 μM) and stained with anti-Tom20 to label mitochondria. Scale bars, 20 μm. (**b**) Quantification of WT and *Atg5*^*−/−*^ MEFs with cleared mitochondria by Tom20 staining after FCCP treatment (*n*=100 cells screened for mitochondria in 3 independent experiments). (**c**) WB time course of Tom20 protein levels in WT and *Atg5*^*−/−*^ MEFs overexpressing Parkin after FCCP treatment (25 μM). (**d**) WB for p62 and Tom20 in *Atg5*^*−/−*^ MEFs overexpressing Ad-βgal or Ad-Parkin after treatment with DMSO or FCCP (25 μM) for 24 h. Band densitometry of p62 (**e**) and Tom20 (**f**) protein levels from **d** (*n*=3, **P<0.01). (**g**) Representative images of *Atg5*^*−/−*^ MEFs infected with mCherry, mCherry-Parkin, mCherry-ParkinR42P or mCherry-ParkinG430D and treated with DMSO or 25 μM FCCP for 24 h. Cells were fixed and stained with anti-Tom20 to label mitochondria. Arrowheads show co-localizing puncta. Scale bars, 20 μm. (**h**) Quantification of Tom20-positive cells after FCCP treatment (*n*=300 cells screened for mitochondria in 3 independent experiments, ***P<0.001). Nuclei were counterstained with Hoechst 33342 (blue). All values are means±s.e.m. from independent experiments. Statistical significance was calculated using analysis of variance followed by Dunnett's test for multiple comparison. Unprocessed original scans of blots are shown in Supplementary Fig. 11.

Recent studies indicate that alternative pathways to traditional autophagy exist that can clear unwanted entire organelles including mitochondria. For instance, autophagy-deficient erythrocytes are still able to eliminate all their organelles including mitochondria during maturation^4^. In addition, during late embryogenesis, epithelial cells covering the anterior surface of the lens differentiate into organelle-free transparent fibre cells during embryogenesis and autophagy-deficient mice are still able to generate normal lens fibres without organelles^12^. To investigate whether Parkin-mediated mitochondrial clearance was altered in autophagy-deficient cells, we used embryonic fibroblasts from *Atg5* knockout mice (*Atg5*^−/−^ MEFs) and their control littermates (wild-type mouse embryonic fibroblasts (WT MEFs))^2^. Consistent with previous studies, treatment of cells with the mitochondrial uncoupler trifluoromethoxy carbonyl cyanide phenylhydrazone (FCCP), led to formation of autophagosomes in WT but not *Atg5*^−/−^ MEFs (Supplementary Fig. 1a,b). Next, we assessed changes in mitochondrial content in response to FCCP treatment in WT and *Atg5*^*−/−*^ MEFs overexpressing YFP-Parkin. FCCP is a potent inducer of Parkin-mediated mitochondrial autophagy^13^. In both WT and *Atg5*^−/−^ MEFs, FCCP treatment led to Parkin translocation and subsequent clearance of mitochondria (Fig. 1a). Intriguingly, the rate of mitochondrial clearance, as assessed by immunfluorescence staining and western blotting (WB) for the mitochondrial protein Tom20, were similar in WT and autophagy-deficient cells (Fig. 1a–c). We confirmed that FCCP-mediated mitochondrial clearance also occurred in primary MEFs deficient for the autophagy-related gene *Atg7* (Supplementary Fig. 1c–f). These results indicate that the traditional *Atg5/7*-dependent autophagy pathway is not required for mitochondrial clearance.

The autophagy adaptor p62/Sqstm1 binds to ubiquitinated mitochondria and is degraded by autophagy along with the mitochondria^14^. Although the *Atg5*^*−/−*^ MEFs are defective in autophagy, both p62/Sqstm1 and Tom20 were still degraded after FCCP exposure (Fig. 1d–f). MEFs, which lack detectable Parkin under normal conditions^15^, overexpressing control vector or non-functional Parkinson's disease-associated mutants ParkinR42P or ParkinG430D were unable to efficiently clear their mitochondria in response to FCCP (Fig. 1g,h). These mutants are defective in the ubiquitin-like and RING2 domains, respectively^16^. Thus, these findings indicate that Parkin-mediated ubiquitination is a prerequisite for degradation of depolarized mitochondria in autophagy-deficient cells.

### Endosomes sequester mitochondria via ESCRT complexes

To examine whether *Atg5/7*-independent alternative autophagy contributed to mitochondrial clearance, we examined *Atg5*^*−/−*^ MEFs for the presence of Rab9-positive vesicles^4^. Although the number of Rab9-positive vesicles significantly increased after FCCP treatment, there was little co-localization between Rab9 and mitochondria in these cells (Supplementary Fig. 2a–c). Furthermore, overexpression of Rab9S21N, a dominant-negative mutant of Rab9 (ref. 17), failed to reduce Parkin-mediated mitochondrial clearance in *Atg5*^−/−^ MEFs (Supplementary Fig. 2d,e). Both alternative autophagy and traditional mitophagy pathways require Ulk1 (refs 4, 18). We confirmed that neither Ulk1 nor its homologue Ulk2 are required for Parkin-mediated clearance in response to FCCP treatment. In fact, we observed similar levels of mitochondrial clearance in WT, *Ulk1*^*−/−*^, *Ulk2*^*−/−*^ and *Ulk1/2*^*−/−*^ MEFs overexpressing Parkin (Supplementary Fig. 3a–d). Thus, these results confirm that traditional and alternative autophagy are not required for Parkin-mediated mitochondrial clearance in *Atg5*^*−/−*^ MEFs.

**Figure 2:**
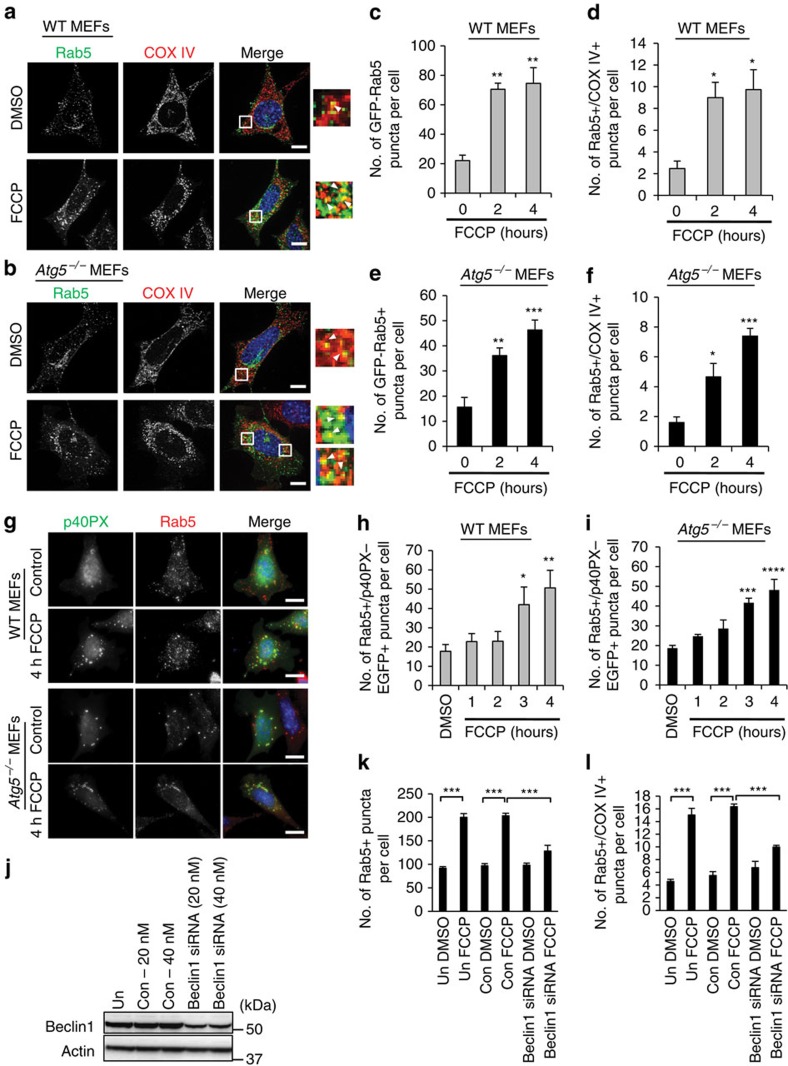
Mitochondrial dysfunction leads to increased number of Rab5-positive endosomes. Representative images of WT (**a**) and *Atg5*^*−/−*^ (**b**) MEFs overexpressing GFP-Rab5, myc-Parkin and stained with anti-COX IV to label mitochondria. Arrowheads show co-localizing puncta. Scale bars, 20 μm. Quantification of GFP-Rab5-positive puncta (**c**) and their co-localization (**d**) with COX IV-labelled mitochondria in WT cells in response to 25 μM FCCP (*n*=45 cells scored for number of puncta in 3 independent experiments, *P<0.05 and **P<0.01 versus 0 h). Quantification of GFP-Rab5-positive puncta (**e**) and their co-localization (**f**) with COX IV-labelled mitochondria in *Atg5*^*−/−*^ cells in response to 25 μM FCCP (*n*=45 cells scored for number of puncta in 3 independent experiments, *P<0.05, **P<0.01 and ***P<0.001 versus 0 h). (**g**) Representative images of WT and *Atg5*^*−/−*^ MEFs overexpressing p40PX-EGFP after DMSO or FCCP treatment (25 μM). Cells were stained with anti-Rab5 to label early endosomes. Scale bars, 20 μm. (**h**,**i**) Quantification of puncta positive for p40PX-EGFP and Rab5 in WT (**h**, *n*=40 cells scored for number of puncta in 4 independent experiments, *P<0.05, **P<0.01, ***P<0.001 and ****P<0.0001 versus DMSO) and *Atg5*^*−/−*^ (**i**, *n*=40 cells scored for number of puncta in 3 independent experiments, *P<0.05 versus DMSO) cells. (**j**) WB for Beclin1 and Actin in *Atg5*^*−/−*^ MEFs transfected with 20 or 40 nM Beclin1 siRNA for 96 h. Quantification of Rab5-positive puncta (**k**) and their co-localization (**l**) with COX IV-labelled mitochondria in untransfected (un), control (con) or 20 nM Beclin1 siRNA-transfected *Atg5*^*−/−*^ cells expressing Parkin in response to DMSO or 25 μM FCCP (*n*=30 cells scored for number of puncta in 4 independent experiments, ***P<0.001). Nuclei were counterstained with Hoechst 33342 (blue). All values are means±s.e.m. from independent experiments. Statistical significance was calculated using analysis of variance followed by Dunnett's test for multiple comparison. Unprocessed original scans of blots are shown in Supplementary Fig. 11.

Next, we sought to ascertain the mechanism of the autophagy-independent mitochondria clearance. The endosomal–lysosomal degradation pathway plays a role in cellular quality control by degrading damaged or excess plasma membrane proteins^19^. However, it is currently unknown whether this pathway also participates in the degradation of entire organelles such as mitochondria. The small GTPase Rab5 is present on the early endosomes and is a critical regulator of their biogenesis and function^20^. We discovered that mitochondrial depolarization with FCCP led to a rapid increase in the number of Rab5-positive early endosomes in WT and *Atg5*^−/−^ MEFs, and that a significant number of these endosomes co-localized with mitochondria after treatment (Fig. 2a–f). Another defining characteristic of early endosomes is the generation of the lipid phosphatidylinositol 3-phosphate (PI(3)P) at the membrane by the Vps34 PI3K complex^21^. Several critical Rab5-interacting proteins bind to PI(3)P at the endsosome^22^. To monitor activity of the Vps34 PI3K complex on Rab5-positive endosomes, we overexpressed a green fluorescent protein (GFP)-conjugated PX domain, which specifically binds PI(3)P at the early endosomes^23^. We observed an increase in the co-localization between p40PX-EGFP and Rab5 in both WT and *Atg5*^*−/−*^ MEFs (Fig. 2g–i) after FCCP treatment. These findings suggest that endosomal activity and sequestration of dysfunctional mitochondria into endosomes occur even in cells with intact autophagy.

There is cross-talk between the various degradation pathways and it is possible that some of the autophagy molecules might also function to regulate the endosome–lysosomal degradation pathway. Beclin1 is a known component of the Vps34 PI3K complex and there is increased interaction between p40PX-EGFP and Beclin1 in both WT and *Atg5*^*−/−*^ MEFs after FCCP treatment (Supplementary Fig. 4a–c). We also observed a significant increase in Beclin1 and Rab5 co-localization in *Atg5*^*−/−*^ MEFs in response to FCCP treatment (Supplementary Fig. 4d–f), suggesting that active Beclin1 is found on the early endosomes. Moreover, silencing of Beclin1 suppressed the increase in Rab5-positive endosomes and the co-localization between Rab5 and mitochondria in *Atg5*^*−/−*^ MEFs (Fig. 2j–l and Supplementary Fig. 4g). We confirmed that *Ulk1/2* deficiency had no effect on early endosomal activity in response to FCCP treatment (Supplementary Fig. 3e,f). Thus, our data indicate that Rab5-mediated mitochondrial clearance requires Beclin1, but not Ulk1/2 or Rab9.

**Figure 3:**
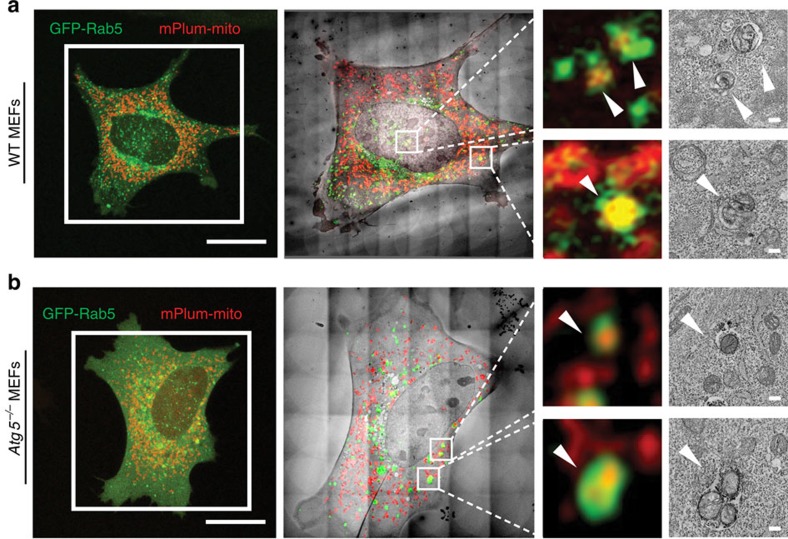
Mitochondria are sequestered inside Rab5-positive endosomes. (**a**) Confocal microscopy of WT (**a**) and *Atg5*^*−/−*^ (**b**) MEFs transfected with GFP-Rab5, VHH-APEX2, mPlum-mito3 and HA-Parkin, and treated with DMSO or FCCP (25 μM) for 4 h. Scale bars, 20 μm. Overlay of confocal inset with electron microscopy of the same cell. Confocal images and electron micrographs of co-localizing puncta (boxes) are shown enlarged and indicated with arrowheads. Note: first inset in **a** is found adjacent to the nuclear envelope, but within the cytosol. Scale bars, 200 nm.

To further confirm that mitochondria were internalized into Rab5-positive endosomes, we performed correlated light and three-dimensional (3D) electron tomography. We observed labelled mitochondria (mPlum-mito3) co-localizing with GFP-Rab5 positive early endosomes after FCCP treatment by confocal microscopy. These co-localization events were confirmed at high resolution by correlated electron microscopy (EM) using a nanobody against GFP fused to APEX2 (ref. 24) (Fig. 3a,b and Supplementary Fig. 5) and subsequent tomographic analysis (Supplementary Movies 1 and 2). We found several instances within each cell type of the entire mitochondria contained in intraluminal vesicles inside single-membrane endosomes. The electron tomographic volumes confirmed the presence of mitochondria fully encased within single-membrane Rab5-positive endosomes. We confirmed that FCCP treatment resulted in engulfment of depolarized mitochondria into Rab5-positive vesicles (Supplementary Fig. 6a). Moreover, at these early time points, within 8 h, there is negligible cell death in response to the FCCP exposure (Supplementary Fig. 6b). Therefore, it is unlikely that these mitochondria within early endosomes are a result of endocytosis from neighbouring dead cells.

**Figure 4:**
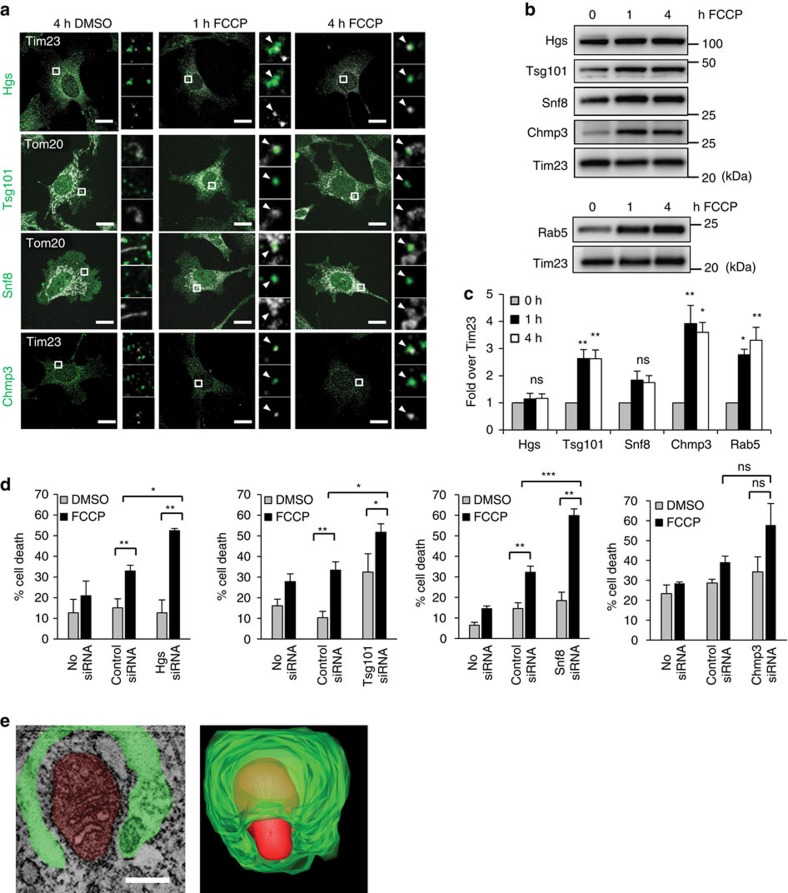
ESCRT complexes participate in sequestration of mitochondria. (**a**) Confocal maximal image projections of *Atg5*^*−/−*^ MEFs infected with mCherry-Parkin and treated with DMSO or 25 μM FCCP for the indicated time. Cells were stained with anti-Tim23 or anti-Tom20 (white) to label mitochondria, and for the indicated ESCRT protein (green). Enlarged boxes are single optical slices with arrowheads showing co-localization. Scale bars, 20 μm. (**b**) Representative WB of isolation of the mitochondrial enriched heavy membrane fraction after 0, 1 or 4 h of 25 μM FCCP treatment in *Atg5*^*−/−*^ MEFs overexpressing Parkin. Tim23 is shown as a mitochondrial loading control. (**c**) Band densitometry of protein levels from **b** (*n*=3, *P<0.05 and **P<0.01; ns=not significant versus 0 h FCCP). (**d**) Quantification of cell death. *Atg5*^*−/−*^ MEFs were exposed to DMSO or FCCP (25 μM) for 24 h after siRNA knockdown and then stained with Po-Pro-3. (*n*=200 screened for cell death in 3 independent experiments, *P<0.05, **P<0.01, ***P<0.001; ns=not significant). (**e**) Single slice of an electron tomogram showing mitochondria (red) partially sequestered by an early endosome (green) in *Atg5*^*−/−*^ MEFs (left). Scale bar, 200 nm. The 3D reconstructed model of mitochondria (red) and endosome (green, right). Movie of the full tomogram of this puncta can be found in Supplementary Movie 2. All values are means±s.e.m. from independent experiments. Statistical significance was calculated using analysis of variance followed by Dunnett's test for multiple comparison. Unprocessed original scans of blots are shown in Supplementary Fig. 11.

Finally, we investigated the mechanism by which mitochondria are sequestered by early endosomes. The ESCRT complexes bind to ubiquitinated cargo and pull them into the endosome through a process of invagination and scission^25^. We found co-localization between mitochondria and proteins in the various ESCRT complexes, Hgs (ESCRT-0), Tsg101 (ESCRT-I), Snf8 (ESCRT-II) and Chmp3 (ESCRT-III) after treatment with FCCP in *Atg5*^*−/−*^ MEFs (Fig. 4a). Furthermore, analysis of the heavy membrane fraction, which is enriched in mitochondria, confirmed a significant increase in several of these ESCRT proteins and Rab5 after FCCP treatment (Fig. 4b,c). As the centrifugation force used to pellet the heavy membrane fraction is too low to spin down isolated early endosomes^26^, this suggests the ESCRT proteins and Rab5 are associated with mitochondria. In addition, knockdown of Hgs, Tsg101 and Snf8 proteins led to decreased mitochondrial clearance and increased cell death in response to FCCP (Supplementary Fig. 7 and Fig. 4d). Damaged mitochondria, if not cleared, can cause cell death through release of pro-death factors and reactive oxygen species^3^,^27^. Thus, reduced removal of mitochondria in cells with ESCRT protein knockdown might be contributing to the enhanced FCCP-mediated cell death. Interestingly, knockdown of Chmp3 (ESCRT-III) did not lead to reduced mitochondrial clearance or significantly enhance FCCP-mediated cell death. This may be due to incomplete knockdown of Chmp3 or compensation by other subunits in the ESCRT-III complex. Finally, we identified a mitochondrion in the process of being engulfed by an early endosome in an electron tomographic volume (Fig. 4e and Supplementary Movie 2), which morphologically supports the ESCRT model of membrane invagination and subsequent scission.

### Mitochondria-containing endosomes mature and are degraded

**Figure 5:**
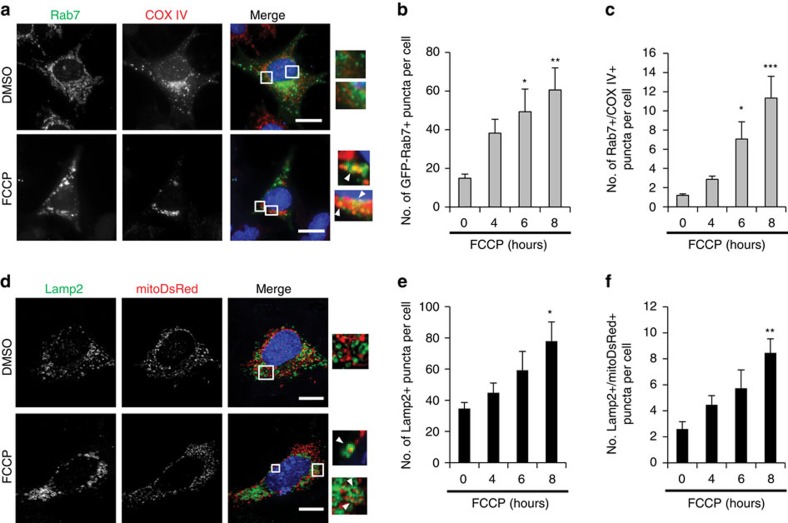
Mitochondria in late endosomes are delivered to lysosomes. (**a**) Representative images of *Atg5*^*−/−*^ MEFs overexpressing GFP-Rab7, HA-Parkin and stained with anti-COX IV to label mitochondria. Arrowheads show co-localizing puncta. Scale bars, 20 μm. (**b**,**c**) Quantification of GFP-Rab7-positive puncta (**b**) and their co-localization (**c**) with COX IV-labelled mitochondria in *Atg5*^*−/−*^ MEFs in response to 25 μM FCCP (*n*=45 cells scored for number of puncta in 3 independent experiments, *P<0.05, **P<0.01 and ***P<0.001 versus 0 h). (**d**) Representative images of cells overexpressing mitoDsRed, HA-Parkin and stained with anti-Lamp2 to label lysosomes. Arrowheads show co-localizing puncta. Scale bars, 20 μm. (**e**,**f**) Quantification of Lamp2-positive puncta (**e**) and their co-localization (**f**) with mitoDsRed in *Atg5*^*−/−*^ MEFs in response to 25 μM FCCP (*n*=40 cells scored for number of puncta in 3 independent experiments, *P<0.05 and **P<0.01 versus 0 h). Nuclei were counterstained with Hoechst 33342 (blue). All values are means±s.e.m. from independent experiments. Statistical significance was calculated using analysis of variance followed by Dunnett's test for multiple comparison.

Early endosomes mature into late endosomes before fusing with lysosomes and this maturation involves a switch from Rab5 to Rab7 (ref. 28). We found that there was a significant increase in the number of Rab7-positive endosomes, and that a significant number of mitochondria co-localized with Rab7 in *Atg5*^*−/−*^ MEFs after FCCP treatment (Fig. 5a–c). The late endosomes fuse with lysosomes where the cargo is degraded. In agreement with this, we observed increased lysosomal activity and a significant number of mitochondria inside LAMP2-positive lysosomes in *Atg5*^*−/−*^ MEFs after FCCP treatment (Fig. 5d–f). Overall, these findings suggest that dysfunctional mitochondria are sequestered inside early endosomes, which mature into late endosomes, and are subsequently delivered to lysosomes for degradation.

Mitochondria can modulate their shape by undergoing fusion or fission. Drp1-mediated asymmetrical fission allows for segregation and selective removal of depolarized mitochondrial fragments by autophagy^29^. However, inhibiting Drp1-mediated mitochondrial fission by overexpressing the dominant-negative Drp1K38E did not affect Parkin-mediated mitochondrial clearance in *Atg5*^*−/−*^ MEFs (Supplementary Fig. 8a,b). Similarly, promoting mitochondrial fusion by overexpression of Mfn2 or Mfn2(T111E/S442E), which lacks mitochondrial fusion activity but is a constitutive Parkin receptor^10^,^13^, did not affect Parkin-mediated mitochondrial clearance in response to FCCP treatment in *Atg5*^*−/−*^ MEFs (Supplementary Fig. 8c–f). Thus, our data suggest that Drp1-mediated mitochondrial fission is not required for clearance of mitochondria by Parkin in *Atg5*^*−/−*^ MEFs. In fact, Drp1-deficient cells have been previously reported to undergo mitochondrial loss^30^, suggesting that there is an alternative mechanism by which mitochondria can fragment to release them from the network and facilitate their clearance. Whether this mechanism is specific for mitochondrial clearance or is involved in the general homeostasis of mitochondria remains to be seen.

### Early endosomal pathway is activated before autophagy

**Figure 6:**
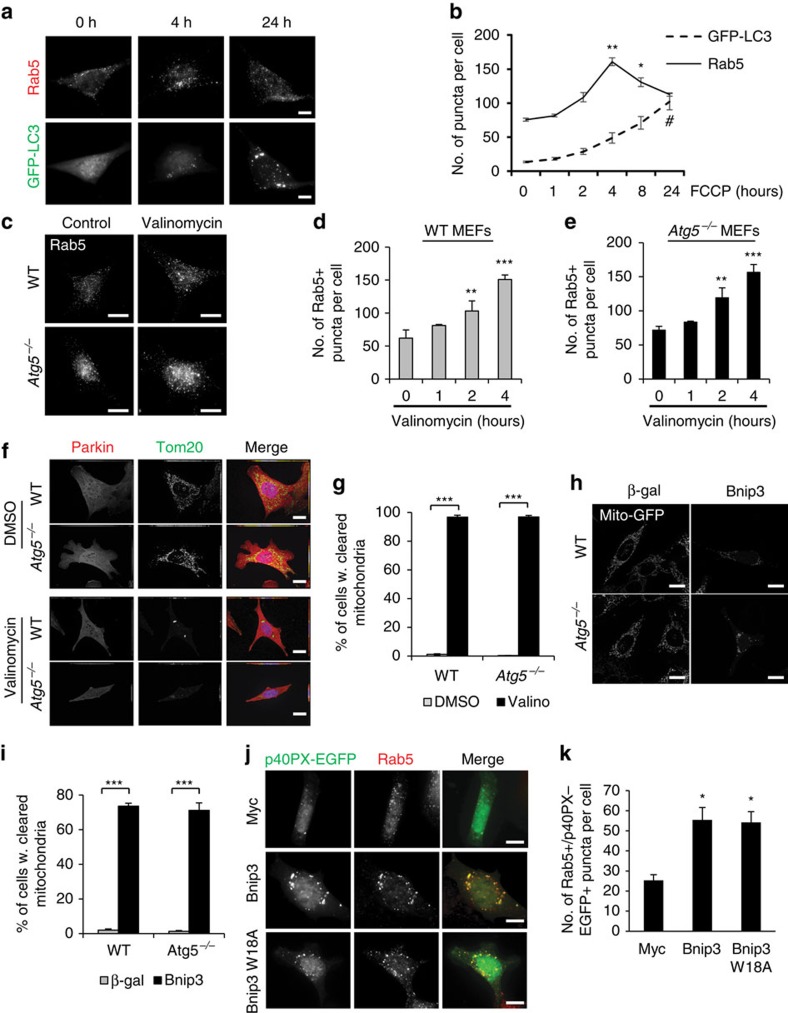
Mitochondrial stress leads to rapid increase in Rab5-positive endosomes. (**a**) Representative images of WT MEFs overexpressing GFP-LC3, treated with 25 μM FCCP for the indicated time and stained with anti-Rab5. Scale bars, 20 μm. (**b**) Quantification of Rab5 and GFP-LC3-positive vesicles in WT cells (*n*=40 cells scored for number of puncta in 3 independent experiments, *P<0.05 and **P<0.01 versus 0 h for Rab5, #*P*<0.05 versus 0 h for GFP-LC3). (**c**) Representative images of WT and *Atg5*^*−/−*^ MEFs infected with mCherry-Parkin and treated with DMSO or 10 μM valinomycin for 4 h. After treatment, cells were fixed and stained with anti-Rab5. Scale bars, 20 μm. (**d**,**e**) Quantification of Rab5-positive vesicles in WT (**d**) and *Atg5*^*−/−*^ (**e**) cells (*n*=30 cells scored for number of puncta in 3 independent experiments, **P<0.01 and ***P<0.001). (**f**) Representative images of WT and *Atg5*^*−/−*^ MEFs infected with mCherry-Parkin and treated with DMSO or 10 μM valinomycin for 24 h. After treatment, cells were fixed and stained with anti-Tom20. Scale bars, 20 μm. (**g**) Quantification of mitochondrial clearance in response to valinomycin (*n*=200 cells screened for mitochondria in 3 independent experiments, ***P<0.001 versus DMSO). (**h**) Representative images of mitochondrial clearance in WT or *Atg5*^*−/−*^ MEFs infected with mito-GFP plus β-gal or Bnip3 for 24 h. Scale bars, 20 μm. (**i**) Quantification of mitochondrial clearance in response to Bnip3 (*n*=100 cells screened for mitochondria in 3 independent experiments, ***P<0.001 versus β-gal). (**j**) Representative images of *Atg5*^*−/−*^ MEFs transfected with myc, Bnip3 or Bnip3W18A plus p40PX-EGFP for 24 h and stained with anti-Rab5. Scale bars, 10 μm. (**k**) Quantification of puncta positive for p40PX-EGFP and Rab5 in *Atg5*^*−/−*^ cells (*n*=35 cells scored for number of puncta in 4 independent experiments, *P<0.05 versus myc). Nuclei were counterstained with Hoechst 33342 (blue). All values are means±s.e.m. from independent experiments. Statistical significance was calculated using analysis of variance followed by Dunnett's test for multiple comparison.

Next, we examined whether the activation of the autophagy and endosomal pathways was simultaneous or sequential in WT cells. Interestingly, we observed a rapid, but transient, increase in the number of Rab5-positive endosomes in response to FCCP treatment, whereas the number of autophagosomes increased progressively but more slowly over time (Fig. 6a,b). Thus, the endosomal pathway might be activated as a first response to clear damaged mitochondria.

We also confirmed that degradation of mitochondria by the endosomal pathway was not limited to FCCP treatment. FCCP depolarizes mitochondria by increasing membrane permeability to H^+^, whereas valinomycin depolarizes mitochondria by permeabilizing the membrane to K^+^. We found that valinomycin treatment led to a significant increase in Rab5-positive puncta, in both WT and *Atg5*^*−/−*^ MEFs (Fig. 6c–e). Valinomycin treatment also resulted in clearance of mitochondria in both WT and *Atg5*^*−/−*^ MEFs in the presence of Parkin (Fig. 6f,g). Mitochondrial clearance can also be directly mediated by mitophagy receptors such as Nix and Bnip3 (ref. 31). These proteins localize to the outer mitochondrial membrane where they induce mitochondrial dysfunction and clearance. We found that overexpression of Bnip3 alone led to efficient clearance of mitochondria in both WT and *Atg5*^*−/−*^ MEFs (Fig. 6h,i). We also confirmed that overexpression of Bnip3 and Bnip3W18A, a mutant unable to dock mitochondria to autophagosomes^32^, led to increased co-localization between p40PX-EGFP and Rab5 in *Atg5*^*−/−*^ MEFs (Fig. 6j,k). Bnip3 is regulated by hypoxia and we confirmed that exposure to hypoxia resulted in significant mitochondrial clearance in *Atg5*^*−/−*^ MEFs by 24 h (Supplementary Fig. 9a). Also, overexpression of the Rab5 dominant negative, Rab5S34N, led to a significant increase in hypoxia-mediated cell death in *Atg5*^*−/−*^ MEFs (Supplementary Fig. 9b). In contrast, DNA damage by Actinomycin D, an inhibitor for RNA synthesis that induces apoptosis^33^, did not lead to an increase in Rab5-positive early endosomes (Supplementary Fig. 9c,d) and did not promote mitochondrial clearance in WT and *Atg5*^*−/−*^ MEFS (Supplementary Fig. 9e,f). These results indicate that mitochondrial stress, but not DNA damage, leads to activation of the endosomal pathway, which correlates with increased mitochondrial clearance.

### Endosomal removal of mitochondria diminishes cell death

**Figure 7:**
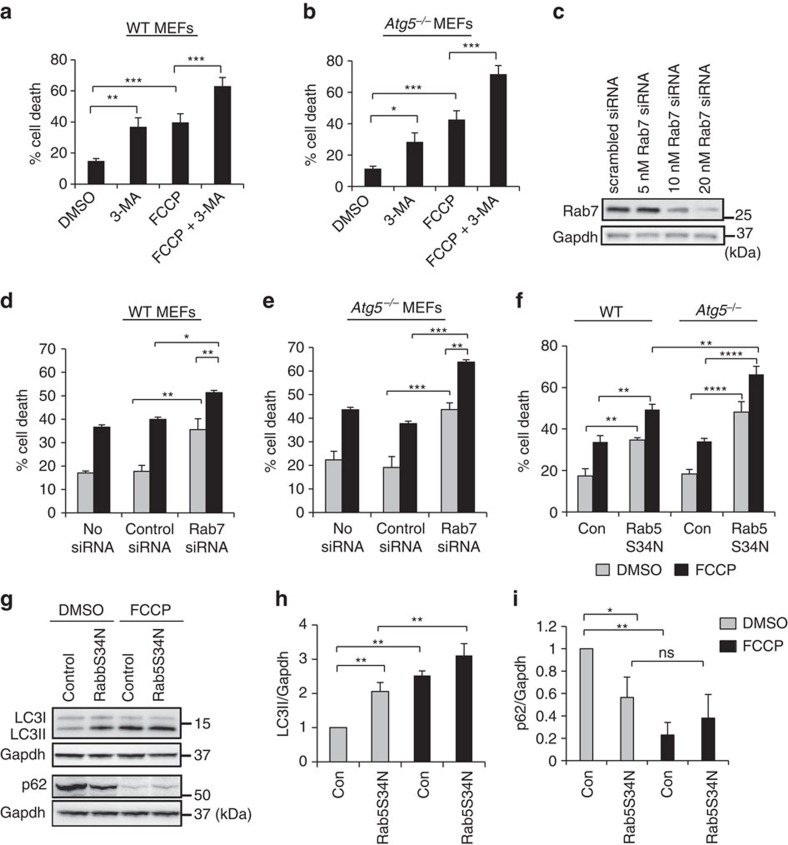
Abrogation of the endosomal–lysosomal degradation pathway increases susceptibility to cell death. (**a**,**b**) Quantification of cell death in response to FCCP +/− 3-MA treatment. WT (**a**) or *Atg5*^*−/−*^ (**b**) MEFs expressing Parkin were incubated with 5 mM 3-MA and DMSO or FCCP (25 μM) for 24 h. Cell death was assessed by Yo-Pro-1 uptake (*n*=350 cells screened for cell death in 3 independent experiments, *P<0.05, **P<0.01 and ***P<0.001). (**c**) WB of Rab7 in *Atg5*^*−/−*^ MEFs after siRNA transfection. (**d**,**e**) Quantification of cell death in WT (**d**) or *Atg5*^*−/−*^ (**e**) cells exposed to DMSO or FCCP (25 μM) for 24 h after siRNA knockdown and then stained with Yo-Pro-1 (**d**: *n*=470, **e**: *n*=175 cells, screened for cell death in 3 independent experiments, *P<0.05, **P<0.01 and ***P<0.001). (**f**) Quantification of cell death in WT and *Atg5*^*−/−*^ MEFs infected with myc-Parkin and βgal (con) or Rab5S34N. Cells were exposed to DMSO or FCCP (25 μM) for 24 h. Cell death was assessed by Po-Pro-3 uptake (*n*=160 cells screened for cell death in 4 independent experiments, **P<0.01 and ****P<0.0001). (**g**) WB for LC3 and p62 levels in WT MEFs expressing Parkin infected with β-gal (control) or Rab5S34N and treated with DMSO or FCCP (25 μM) for 4 h. (**h**,**i**) Band densitometry of LC3II (**h**) and p62 (**i**) protein levels (*n*=3, *P<0.05 and **P<0.01, ns=not significant). All values are means±s.e.m. from independent experiments. Statistical significance was calculated using analysis of variance followed by Dunnett's test for multiple comparison. Unprocessed original scans of blots are shown in Supplementary Fig. 11.

To explore the functional importance of the endosomal degradation pathway, we used 3-methyladenine (3-MA) to pharmacologically inhibit the endosomal–lysosomal pathway. 3-MA is a PI3K inhibitor that inhibits Vps34 and disrupts formation of both endosomes and autophagosomes^34^. We found that inhibition of Vps34 by 3-MA led to increased susceptibility to FCCP-mediated cell death in WT and *Atg5*^*−/−*^ MEFs (Fig. 7a,b). In addition, knockdown of the late endosome protein Rab7 led to increased FCCP-mediated cell death in WT and *Atg5*^*−/−*^ MEFs (Fig. 7c–e). Rab5 exists as three isoforms with overlapping functions and all three isoforms are expressed in MEFs (Supplementary Fig. 10a,b). The dominant-negative Rab5S34N targets all three isoforms of Rab5 and effectively reduced FCCP-mediated mitochondrial clearance in cells (Supplementary Fig. 10d,e). Overexpression of Rab5S34N also caused increased FCCP-mediated cell death in both WT and *Atg5*^*−/−*^ MEFs. Consistent with the previous experiment, the *Atg5*^*−/−*^ MEFs were significantly more susceptible to Rab5 inhibition than WT cells (Fig. 7f). Interestingly, we observed that inhibition of the endosomal pathway alone led to an increase in cell death in both WT and *Atg5*^*−/−*^ MEFs, confirming the importance of the endosomal pathway in cellular homeostasis and in clearing damaged mitochondria. However, because the endosomal pathway is involved in many cellular processes, we cannot rule out the possibility that these results are influenced by other stresses induced by inhibition of the endosomal pathway.

To determine why WT cells were less susceptible to abrogation of the endosomal pathway, we examined the relationship between the autophagy and endosomal pathways in WT cells. Interestingly, inhibition of the endosomal pathway led to increased autophagic activity under both baseline conditions and after stress (Fig. 7g–i). Although abrogating Rab5 alone led to increased autophagic activity, we did not observe an increase in mitophagic activity under baseline conditions (Supplementary Fig. 10f,g). Assessing mitophagy at later time points is challenging due to the amount of cell death induced by FCCP in cells overexpressing Rab5S34N. Thus, this suggests that there is cross-talk between the two pathways, and that the autophagy pathway can potentially compensate when the endosomal pathway is impaired. However, further studies are needed to examine the relationship between these pathways.

**Figure 8:**
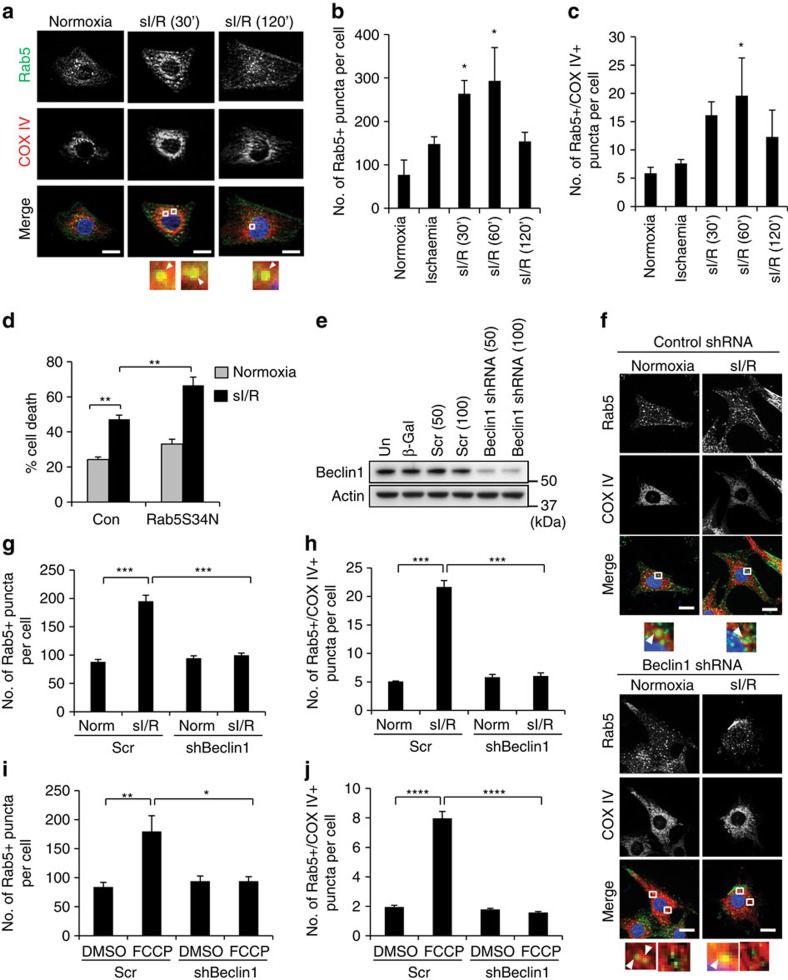
Activation of endosomal-mediated mitochondrial degradation in cardiac myocytes. (**a**) Representative images of neonatal myocytes subjected to normoxia or sI/R. After treatment, cells were fixed and stained with anti-Rab5 and anti-COX IV. Arrowheads show co-localizing puncta. Scale bars, 10 μm. (**b**,**c**) Quantification of Rab5-positive puncta (**b**) and their co-localization (**c**) with COX IV-labelled mitochondria in neonatal cardiac myocytes (*n*=36 cells scored for number of puncta in 3 independent experiments, *P<0.05 versus normoxia). (**d**) Quantification of cell death. Cells were exposed to normoxia or 2 h of simulated ischaemia plus 8 h of reperfusion. Cell death was assessed by propidium iodide staining (*n*=170 cells screened for cell death in 3 independent experiments, **P<0.01). (**e**) WB of Beclin1 knockdown in myocytes. (un=uninfected, scr=scramble shRNA, parentheses indicate MOI) (**f**) Representative images of neonatal myocytes after Beclin1 knockdown. After treatment, cells were stained with anti-Rab5 and anti-COX IV. Scale bars, 10 μm. Arrowheads show colocalization. Enlarged boxes without arrowheads show non-co-localizing puncta. (**g**,**h**) Quantification of Rab5-positive puncta (**g**) and their co-localization (**h**) with COX IV-labelled mitochondria in neonatal cardiac myocytes. Cells were treated with scramble (scr) or Beclin1 shRNA (MOI=50) and subjected to normoxia or 2 h of simulated ischaemia plus 60 min of reperfusion before fixation and staining with anti-Rab5 and anti-COX IV (*n*=30 cells scored for number of puncta in 3 independent experiments, ***P<0.001). (**i**,**j**) Quantification of Rab5-positive puncta (**i**) and their co-localization with COX IV-labelled mitochondria (**j**) in neonatal cardiac myocytes. Cells were subjected to FCCP (10 μM) for 6 h before fixation and staining with anti-Rab5 and anti-COX IV (*n*=30 cells scored for number of puncta in 3 independent experiments, *P<0.05, **P<0.01 and ****P<0.0001). Nuclei were counterstained with Hoechst 33342 (blue). All values are means±s.e.m. from independent experiments. Statistical significance was calculated using analysis of variance followed by Dunnett's test for multiple comparison. Unprocessed original scans of blots are shown in Supplementary Fig. 11.

Maintaining a functional population of mitochondria is critical for cardiac myocyte function. They are highly active cells that require large amounts of energy supplied by mitochondrial oxidative phosphorylation. As myocytes are postmitotic, it is critical for these cells to efficiently remove dysfunctional mitochondria before they can cause harm. Therefore, we investigated whether the endosomal pathway contributes to clearing damaged mitochondria in myocytes. Myocardial ischaemia/reperfusion is associated with mitochondrial damage and activation of mitophagy^35^. Interestingly, we found that simulated ischaemia/reperfusion (sI/R) led to a rapid transient increase in the number of Rab5-positive endosomes on reperfusion (Fig. 8a,b). sI/R has many effects on the cell, so it is currently unclear whether mitochondrial dysfunction is the only trigger for increased endosome formation. However, we observed that many of the endosomes co-localized with mitochondria in the myocytes (Fig. 8c). In addition, all three isoforms of Rab5 are expressed in the heart (Supplementary Fig. 10c) and overexpression of Rab5S34N to inhibit Rab5 activity led to increased sI/R-mediated cell death (Fig. 8d). Finally, we confirmed that Beclin1 regulates endosomal activity in myocytes in response to stress. Knockdown of Beclin1 using Ad-shRNA completely abrogated the increase in Rab5-positive endosomes and their co-localization with mitochondria in myocytes in response to sI/R or FCCP treatment (Fig. 8e–j). These data confirm that Rab5 and the endosomal pathway also contribute to clearing impaired mitochondria in cardiac myocytes.

## Discussion

**Figure 9:**
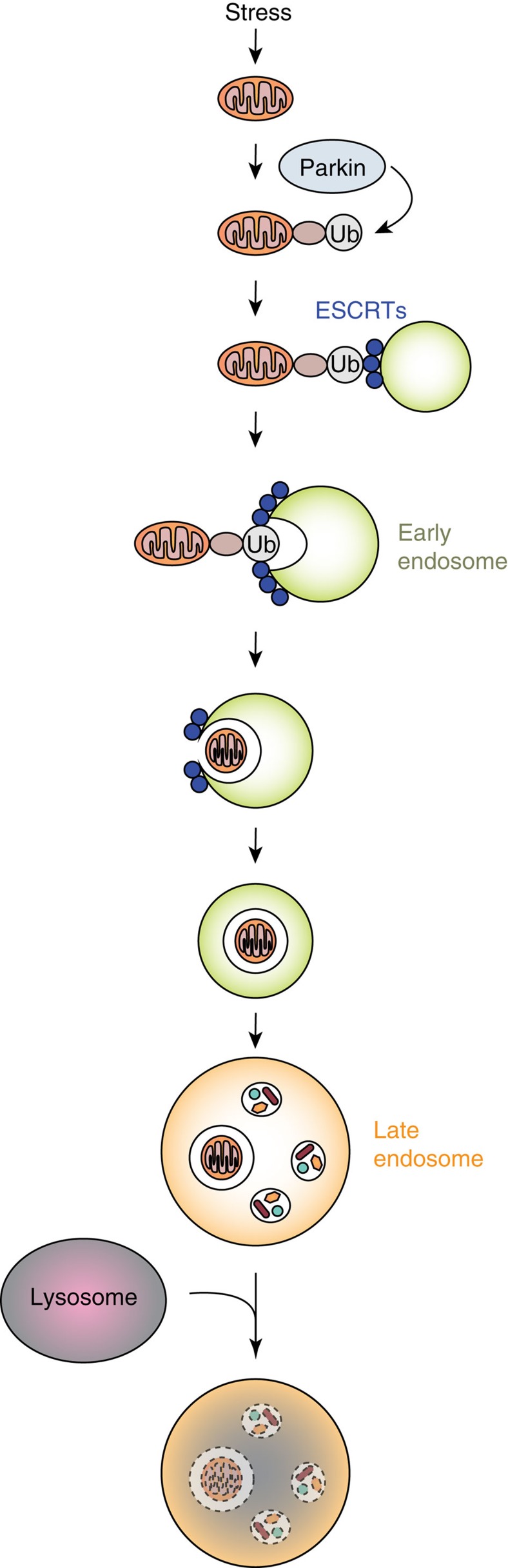
Model of endosomal sequestration and degradation of a damaged mitochondrion. Stress, such as FCCP or sI/R, induces Parkin recruitment and ubiquitination of proteins in the outer mitochondrial membrane. The ubiquitinated mitochondrion is captured by the ESCRT complexes on the early endosome. The ESCRT machinery induces invagination and subsequent scission of the endosome membrane, leading to internalization of the mitochondrion in the lumen of the endosome.

For a long time, it was believed that autophagy was the only pathway involved in degrading organelles such as mitochondria. In this study, we have uncovered a previously unidentified mechanism of mitochondrial clearance that exists in cells. Our data suggest that depolarized mitochondria can be sequestered in single-membrane Rab5-positive endosomes via the ESCRT machinery, and that these vesicles mature into Rab7-positive late endosomes before being delivered to lysosomes for degradation (Fig. 9). This pathway is also activated in cells with functional autophagy and disrupting the endosomal pathway impaired mitochondrial clearance and increased the cells' susceptibility to death, confirming the physiological importance of this pathway in cells.

Our data suggest that the delivery of mitochondria into endosomes is distinct from autophagic engulfment. The 3D reconstruction of electron tomographic volumes clearly shows that mitochondria are engulfed via a process involving invagination of the vesicle membrane. Our data also show that mitochondria inside the vesicles are surrounded by intraluminal membranes. These are produced by the invagination and scission of the limiting membrane and is reminiscent of ESCRT-dependent processes. The ESCRT complexes are responsible for cargo capturing and sorting, as well as membrane invagination and scission^36^. This is in contrast to autophagy, where a cup-shaped phagophore membrane binds to ubiquitinated cargo via autophagy receptors such as p62 and Nbr1, and then encloses around the cargo to form a double membraned autophagosome^3^.

Our data further suggest that degradation of mitochondria in response to FCCP-mediated depolarization in both WT and *Atg5*^*−/−*^ is dependent on the presence of Parkin. Parkin is an E3 ubiquitin ligase that ubiquitinates proteins in the outer mitochondrial membrane^3^. Both autophagy receptors, such as p62 and Nbr1, and ESCRT-0 bind ubiquitinated cargo to be degraded^3^,^36^. This suggests that the primary function of Parkin is to label mitochondria for degradation. In contrast to our study, Narendra *et al*.^11^ reported that Parkin did not promote clearance in *Atg5*^*−/−*^ MEFs in response to the mitochondrial uncoupler CCCP. We believe this discrepancy is due to differences in the dose. This study used a lower dose than us and they also reported much lower levels of Parkin-mediated mitochondrial clearance in WT MEFs than our study. Clearly, a lower dose will lead to reduced and/or a slower rate of clearance.

A recent study reported that mitochondria can also be directly delivered to lysosomes via catalytically inactivated glyceraldehyde 3-phosphate dehydrogenase^5^. An impairment in this process, which is also known as microautophagy^37^, plays a role in Huntington's disease^5^. When there is limited damage, the mitochondrion can repair itself by eliminating damaged lipids and proteins via mitochondrial-derived vesicles (MDVs) that bud off and then fuse with lysosomes^38^. Interestingly, it has been shown that transcellular degradation of mitochondria exists in certain areas in the central nervous sytem where mitochondria are internalized and degraded by adjacent cells, more specifically astrocytes^39^. Thus, it is clear that multiple quality control mechanisms exist to ensure rapid clearance of dysfunctional mitochondria. The redundancy ensures that mitochondria can be removed before they cause unnecessary cell death even if one of the pathways is impaired or not functioning properly. Our data suggest that the endosomal–lysosomal system is but one pathway through which mitochondria can clear. Clearly, multiple levels of mitochondrial quality control exist to ensure mitochondrial health, and it is very possible that additional, currently unidentified, pathways of mitochondrial quality control exist in cells.

In addition, our data show that activation of endosomal-mediated mitochondrial clearance precedes activation of the autophagy pathway. We propose that uptake of mitochondria into endosomes acts as a first line of defense to rapidly eliminate dysfunctional mitochondria. Early endosomes are continuously synthesized and exist in a network in cells^20^, whereas autophagosomes are synthesized *de novo* on demand^40^. Therefore, dysfunctional mitochondria can be more rapidly sequestered by the early endosomes. However, it is likely to be that if the number of damaged mitochondria exceeds the capacity of endosomal-mediated degradation, then the autophagy pathway is also activated. A similar coordination and sequential activation has been observed for the traditional autophagy and chaperon-mediated autophagy pathways in response to stress such as starvation and oxidative stress^41^. Traditional autophagy is rapidly activated in response to stress but then there is a switch to chaperon-mediated autophagy if the stress is prolonged. Future studies need to determine the context and timing of activation of these various pathways in cells. Our data also show that autophagic activity is increased when Rab5 activity is abrogated, suggesting that autophagic degradation is compensating when endosomal activity is impaired.

It is clear that many of the proteins involved in cellular quality control regulate multiple quality control pathways. For instance, Beclin1 appears to be a central regulator of the endosomal, traditional and alternative autophagy pathways^4^,^42^. Similarly, the Rab proteins are well-known for their roles in regulating endosomal trafficking, but recent studies have found that some of the Rab proteins can also regulate autophagy^43^,^44^. Parkin is known to mark mitochondria for autophagic degradation and here we demonstrate that Parkin also labels mitochondria for endosomes. Both endosomes and autophagosomes recognize ubiquitinated targets^45^. Parkin is also involved in the formation of a subset of MDVs that are destined for delivery to late endosomes/lysosomes^46^. Although the Rab5 pathway described in our study and formation of MDVs both use the endosomal–lysosomal degradation pathway and are independent of Atg5, these are clearly two distinct mechanisms of mitochondrial quality control. First, we observe that entire mitochondria are sequestered inside large (∼500 nm) single-membrane Rab5-positive early endosomes. In contrast, MDVs are small vesicles (70–150 nm) containing select mitochondrial proteins and lipids that bud off the outer mitochondrial membrane mitochondria, which are then delivered directly to LAMP1-positive late endosomes/lysosomes. Finally, our data show that the mitochondria are captured by the early endosomes through ESCRT-mediated membrane invagination and subsequent scission, whereas MDVs directly fuse with late endosomes/lysosomes via Stx17 and SNARE complexes to deliver their content^47^. Thus, it is likely to be that formation of MDVs occurs when there is limited damage in an attempt to avoid degradation of the entire mitochondrion. However, when the damage is too extensive, then the entire mitochondrion is sequestered inside a Rab5-positive early endosome.

In addition, there are receptors on mitochondria that directly target them to autophagosomes bypassing the need for Parkin. Interestingly, we found that one of these receptors, Bnip3, induced increased endosomal activity in autophagy-deficient MEFs, which correlated with mitochondrial clearance. However, whether Bnip3 and other known mitophagy receptors use endosomal-mediated degradation of mitochondria and the ESCRT machinery needs to be further investigated. There is some evidence in the literature that the ESCRT machinery can participate in autophagic degradation of cargo. It was recently reported that the fission yeast Nbr1, a homologue of the mammalian autophagy receptor NBR1, facilitated the transport of two cytosolic hydrolases into vacuoles via the ESCRT proteins^48^. In addition, the ESCRT machinery has been reported to participate in delivering autophagic cargo into multivesicular bodies in a process known as microautophagy^49^. In this process, soluble proteins containing the KFERQ-like targeting motif are delivered into late endosomes/multivesicular bodies in a manner dependent on ESCRTs and the chaperone protein Hsc70 (ref. 49). Overall, this suggests that there is a lot of cross-talk and overlap between the various degradation pathways.

Although overexpression of proteins is very useful tool to study protein function and signalling pathways, there is always the concern that the overexpression interferes with normal cell function. To study Parkin-mediated mitochondrial degradation, we relied on overexpression of Parkin in cells. One advantage is that we can study Parkin and mutants in cells such as MEFs that have no detectable levels of endogenous Parkin. Second, using fluorescently tagged Parkin has allowed us and numerous other investigators^11^,^13^,^50^ to monitor translocation of Parkin to mitochondria in live cells in response to stress. The disadvantages are that to visualize and study Parkin in cells, the protein is expressed at levels that are well above the normal physiological levels in cells and tissues. However, we have confirmed our key findings in neonatal myocytes that express endogenous Parkin. Furthermore, studies have reported that Parkin is significantly upregulated in tissues in response to various stressors^7^,^8^,^51^,^52^. Thus, Parkin overexpression studies are more likely to mimic signalling events in diseases or stressed tissues rather than under baseline conditions. Another limitation of our study is that it is still unclear whether the increase in Rab5+ endosomes observed in response to FCCP treatment is predominantly due to mitochondrial stress or whether signals from dysfunctional late endosomes/lysosomes are also contributing to this increase. Although FCCP is a potent inducer of Parkin-mediated mitochondrial degradation^11^, it can also perturb V-ATPase-mediated acidification of endosomal/lysosomal compartments^53^. These pH changes in turn can act on several pathways, such as TORC1 and AMPK signalling^54^,^55^, which can affect biogenesis of endosomes. For instance, the transcription factor EB (TFEB) regulates expression of genes involved in endosomal and lysosomal biogenesis and activity. Inhibition of lysosomal function was reported to reduce MTORC1-dependent phosphorylation of TFEB, resulting in its nuclear translocation and subsequent increased expression of its target genes^56^. Thus, it is possible that disruption of lysosomal acidification and function by FCCP could contribute to increased endosomal biogenesis via TFEB activation.

In conclusion, our studies have provided insights into a new mitochondrial quality control pathway as shown in our model in Fig. 9. Future studies need to validate this pathway *in vivo* and to investigate the functional relevance of this pathway to human health and in disease. Mitochondrial quality control involves a very complex network of proteins and pathways, many of which have probably not been identified yet. Considering that mitochondrial dysfunction contributes to neurodegenerative and cardiovascular diseases, it is critical to gain increased understanding of the pathways that regulate mitochondrial health. This will ultimately allow for the identification of novel therapeutic targets in these pathways that could lead to effective new treatments for diseases.

## Methods

### Antibodies

The following antibodies were used for immunofluorescence (IF) and WB experiments: Actin (WB 1:2,000; Sigma; A4700), Beclin1 (WB 1:1,000; Santa Cruz; sc-11427), Chmp3 (IF 1:100; WB 1:1,000; Santa Cruz; sc-67228), Gapdh (WB 1:2,000, Genetex; GTX627408), Hgs (IF 1:100; WB 1:1,000; Abcam; ab155539), Lamp2 (IF 1:100; Abcam; ab13524), Lamp2 (WB 1:1,000; Santa Cruz; sc-18822), LC3 (WB 1:1,000; Cell Signaling; 4108), OxPhos Complex IV subunit 1 (IF 1:100; Life Technologies; 459600), p62 (WB 1:1,000; ARP; 03-GP62-C), Rab5 (IF 1:100; WB 1:1,000; Cell Signaling; 3547), Rab7 (WB 1:1,000; Cell Signaling; 9367), Snf8 (IF 1:100; WB 1:1,000; Santa Cruz; sc-390747), Tim23 (IF 1:100; WB 1:1,000; BD Biosciences; 611222), Tom20 (IF 1:200; WB 1:1,000; Santa Cruz; sc-11415), Tsg101 (IF 1:100; WB 1:1,000; Abcam; ab83) and tubulin (WB 1:2,000; Sigma; T6074). Secondary antibodies used were goat anti-mouse or goat anti-rabbit HRP, Alexa Fluor 488 or 594 (Life Technologies).

### Cells and culture conditions

MEFs were maintained in MEF culture media (DMEM (Life Technologies) supplemented with 10% fetal bovine serum (Life Technologies), 100 U ml^−1^ penicillin (Gemini) and 100 μg ml^−1^ streptomycin (Gemini)), and cultured in a 5% CO_2_ atmosphere at 37 °C. WT and *Atg5*^−/−^ MEFs were generously provided by Dr Noboru Mizushima (The University of Tokyo, Japan); primary WT and *Atg7*^*−/−*^ MEFs were kindly provided by Dr Toren Finkel (NIH, USA)^57^; WT and *Ulk1*^−/−^ MEFs were generously provided by Dr Mondira Kundu (St Jude Children's Research Hospital, USA)^18^; *Ulk2*^−/−^ and *Ulk1/2*^−/−^ MEFs were obtained from Cancer Research Technology Ltd. None of these cell lines are listed in the database of commonly misidentified cell lines maintained by ICLAC. Neonatal cardiac myocytes were prepared from 1- to 2-day-old Sprague–Dawley rats by enzymatic digestion of the hearts^58^ and subsequent plating on gelatin-coated Nunc Lab-Tek Chamber Slides (Thermo Scientific) in a 4:1 ratio DMEM (Life Technologies) to M199 (Life Technologies) plus 10% fetal bovine serum (Life Technologies), 100 U ml^−1^ penicillin (Gemini), 100 μg ml^−1^ streptomycin (Gemini) and 100 μM BrdU (Sigma). Myocytes were cultured in serum-free media at 37 °C with 5% CO_2_. Cell lines have not been tested for mycoplasma contamination. sI/R was initiated by incubating myocytes in ischemic buffer (125 mM NaCl, 8 mM KCl, 1.2 mM KH_2_PO_4_, 1.25 mM MgSO_4_, 1.2 mM CaCl_2_, 6.25 mM NaHCO_3_, 20 mM 2-deoxyglucose, 5 mM Na-lactate, 20 mM HEPES pH 6.6) and placing the dishes in hypoxic pouches (GasPak EZ, BD Biosciences), equilibrated to 95% N_2_, 5% CO_2_. Reperfusion was initiated by changing to Krebs–Henseleit buffer (110 mM NaCl, 4.7 mM KCl, 1.2 mM KH_2_PO_4_, 1.25 mM MgSO_4_, 1.2 mM CaCl_2_, 25 mM NaHCO_3_, 15 mM glucose, 20 mM HEPES pH 7.4). Normoxic control cells were maintained in Krebs–Henseleit buffer throughout the experiment. All animal protocols were in accordance with institutional guidelines and approved by the Institutional Animal Care and Use Committee of the University of California, San Diego. Hypoxia experiments were performed by placing cells in hypoxic buffer (MEF culture media as described above, plus 20 mM HEPES pH 7.4 (Gibco)) and placing the dishes in a 5% CO_2_ atmosphere at 37 °C for normoxic controls, or in hypoxic pouches (GasPak EZ, BD Biosciences), equilibrated to 95% N_2_, 5% CO_2_ for hypoxic experimental conditions.

### Transient transfections and siRNA knockdown

Cells were transiently transfected with DNA using Fugene 6 Transfection Reagent (Promega) according to the manufacturer's instructions. Generation of Bnip3W18A was prepared using site directed mutagenesis by PCR with Bnip3 WT as a template^32^. YFP-Parkin was a gift from Richard Youle (Addgene plasmid 23955)^11^; Myc-Parkin and HA-Parkin were gifts from Ted Dawson (Addgene plasmids 17612 and 17613)^59^; EGFP-Rab5S34N was a gift from Qing Zhong (Addgene plasmid 28045)^60^; p40PX-EGFP was a gift from Michael Yaffe (Addgene plasmid 19010)^23^; GFP-Rab9 and GFP-Rab9S21N were gifts from Richard Pagano (Addgene plasmids 12663 and 12664)^17^; mPlum-Mito-3 was a gift from Michael Davidson (Addgene plasmid 55988)^61^; and GFP-Rab5 and GFP-Rab7 were gifts from JoAnn Trejo (UCSD, USA). Beclin1 knockdown experiments were performed by transfecting 20 nM AllStars Negative Control (Qiagen, number SI03650318; seq: 5′-CAGGGTATCGACGATTACAAA-3′) or Beclin 1 small interfering RNA (siRNA) (Sigma, siRNA ID: SASI_Mm01_00048143; seq: 5′-CUGAGAAUGAAUGUCAGAA-3′) with HiPerFect Transfection Reagent (Qiagen) for 96 h, according to the manufacturer's instructions. Rab7 knockdown experiments were performed by transfecting 20 nM Rab7 siRNA SMARTpool (Dharmacon, no. L-040859-02-0005; seqs: 5′-CAGCUGGAGAGACGAGUUU-3′, 5′-CGACAGACUUGUUACCAUG-3′, 5′-GAGCGGACUUUCUGACCAA-3′ and 5′-CAGAAGUGGAACUGUACAA-3′) or AllStars Negative Control siRNA with Dharmafect 4 Transfection Reagent (Dharmacon) for 96 h, according to the manufacturer's instructions. Knockdown of ESCRT proteins was performed by transfecting 20 nM AllStars Negative Control, 80 nM Hgs siRNA SMARTpool (Dharmacon, number L-055516-01-0005, seqs: 5′-CAAGAUACCUCAACCGGAA-3′, 5′-AAGCAUCACUGCCGAGCAU-3′, 5′-CGUACAAUAUGCAGAAUCU-3′ and 5′-AGACAGACUCUCAGCCCAU-3′), 40 nM Tsg101 siRNA (Sigma, siRNA ID: SASI_Mm01_00065312; seq: 5′-GGUACAAUCCCAGUGCGUU-3′), 80 nM Snf8 siRNA SMARTpool (Dharmacon, number L-049190-01-0005; seqs: 5′-UCGGAAUGGAGGUCUGAUA-3′, 5′-GACUGAGUGUGGAGGGGUA-3′, 5′-CAAGAGAUCCGGAAGAAUC-3′, 5′-CUACAUCAGCAGGUGUUAA-3′), or 100 nM Chmp3 siRNA SMARTpool (Dharmacon, no. L-062411-01-0005; seqs: 5′-GUGAAAUGCAGGACAGUUA-3′, 5′-GGGUUAACGUGCUGUGUUG-3′, 5′-UGAGAAGAGUAGAUUGAAU-3′ and 5′-GUACAGAAUGGCUUUGUUA-3′), with HiPerFect Transfection Reagent (Qiagen) for 96 h, according to the manufacturer's instructions.

### Adenoviral infections and shRNA knockdown

Cells were infected with adenoviruses in DMEM+2% heat-inactivated serum for 3 h and rescued with growth media. Experiments were performed 20 h later. Generation of adenoviruses encoding mCherry-Parkin, mCherry-ParkinR42P and mCherry-ParkinG430D were previously described^7^. The Bnip3 adenovirus was previously described^32^. The myc-Parkin, p40PX-EGFP and Drp1K38E adenoviruses were generated using the pENTR directional TOPO cloning kit (Invitrogen) followed by recombination into the pAd/CMV/V5-DEST Gateway vector (Invitrogen). Ad-GFP-Rab5S34N was a gift from Pyong Woo Park (Boston Children's Hospital, USA)^62^; Mfn2WT and Mfn2EE viruses were provided by Gerald W. Dorn II^13^. Ad-Beclin1 short hairpin RNA (shRNA) was provided by Dr Junichi Sadoshima^63^. Knockdown experiments were performed by infecting cells with Ad-U6-scramble-RNAi control (Vector Biolabs, 1122) or Ad-Beclin1 shRNA and experiments were performed 96 h later.

### Cell death assays

Cells infected with Ad-β-gal or Ad-myc-Parkin (multiplicity of infection (MOI): 150) were treated with vehicle or 5 mM 3-methyladenine (Sigma) for 30 min before dimethyl sulfoxide (DMSO; Sigma) or 25 μM FCCP (Sigma) exposure. Cells with Rab7 knockdown were infected with Ad-mCherry-Parkin (MOI: 150) and then re-transfected with control or Rab7 siRNA. Cells overexpressing Ad-GFP or Ad-GFP-Rab5S34N (MOI: 75) plus Ad-myc-Parkin (MOI: 150) were treated with DMSO or 25 μM FCCP for 24 h. Cells with ESCRT protein knockdowns were infected with Ad-mCherry-Parkin (MOI: 75) and then re-transfected with control or ESCRT-specific siRNA for another 48 h. Cells were then treated with DMSO or 25 μM FCCP for 24 h. In hypoxia cell death experiments, cells were infected with Ad-mCherry-Parkin (MOI: 75) and Ad-GFP or Ad-GFP-Rab5S34N (MOI:40), placed in hypoxic buffer and kept at normoxia or hypoxia for 34 h as described above. To assess viability, cells were stained with Yo-Pro-1 (1:1,000; Life Technologies), propidium iodide (1:3,000; Life Technologies) or with Po-Pro-3 (1:1,000; Life Technologies) plus Hoechst 33342 (invitrogen) for 20 min. Cell death was assessed as the number of Yo-Pro-1, propidium iodide or Po-Pro-3-positive cells divided by total number of Hoechst 33342-positive cells as described^64^.

### Immunofluorescence

Cells were fixed with 4% paraformaldehyde (Ted Pella Inc.) in Dulbecco's PBS without calcium chloride and magnesium chloride (PBS) (Gibco, 14200-075), permeabilized with 0.2% Triton X-100 or 0.1% saponin (for Lamp2 staining) in PBS and blocked in 5% normal goat serum. Myocytes were blocked with 5% normal goat serum plus 0.2% Tween20 in PBS (PST). Cells were incubated with primary antibodies (4 °C, overnight) in PBS (MEFs) or PST (myocytes), rinsed with PBS, incubated with secondary antibodies (37 °C, 1 h) and counter-stained with Hoechst 33342. Fluorescence images were captured using a Carl Zeiss Axio Observer Z1 fitted with a motorized Z-stage with a × 63 Plan-Apochromat (oil immersion) objective. *Z*-stacks of red and green fluorescence separated by 0.6 μm along the *z* axis were acquired with an ApoTome using a high-resolution AxioCam MRm digital camera and Zeiss AxioVision 4.8 software (Carl Zeiss). Mitochondrial clearance was scored on a per cell basis. Mitochondrial clearance progresses through a well-documented series of steps including nuclear aggregation before complete removal^65^; thus, cells displaying nuclear aggregation or lack of mitochondria were scored as ‘cleared.' Endosomal and autophagic vesicles in cells were quantified by counting the number of distinct, visible puncta in a maximal projection image of each cell. Co-localization was determined by manually scoring the number of puncta on single *Z*-stack slices that had clear signal in both red and green image channels. All scoring of morphology and puncta counts were scored unblinded with respect to sample identity. Confocal images were captured on a Leica SPE II (Leica) inverted confocal microscope equipped with 405, 488, 561 and 637 nm lasers, with 0.25 μm step size between optical sections. Assessment of mitochondrial depolarization was performed by transfecting cells with HA-Parkin and GFP-Rab5, treating with DMSO or 25 μM FCCP for 4 h and adding MitoTracker Red CMXROS (Thermo Fisher Scientific, M7512) at a final concentration of 250 nM for 20 min before fixation and subsequent staining.

### WB analysis

Cells were lysed in 50 mM Tris-HCl, 150 mM NaCl, 1 mM EGTA, 1 mM EDTA, 1% Triton X-100 and Complete protease inhibitor cocktail (Roche). Lysates were cleared by centrifugation at 20,000 *g* for 20 min at 4 °C. Proteins were separated on NuPAGE Bis-Tris gels (Life Technologies) and transferred to nitrocellulose membranes. Membranes were incubated with the indicated antibodies and imaged with a Bio-Rad ChemiDoc XRS+ imager. Band densitometry quantification was performed using the software Quantity One (Bio-Rad). Unprocessed original scans of blots are shown in Supplementary Fig. 11.

### Isolation of the heavy membrane fraction

*Atg5*^*−/−*^ MEFs were infected with mCherry-Parkin virus (MOI: 75) and were treated with DMSO or 25 μM FCCP for 4 h. Cells were collected using a cell scraper and incubated in isolation buffer (220 mM mannitol, 70 mM sucrose, 1 mM EDTA and 10 mM HEPES at 7.4 pH) for 45 min on ice. Cells were homogenized using an Eppendorf pestle and centrifuged for 10 min at 1,000 g to pellet unbroken cells. The supernatant was collected and centrifuged for 15 min at 14,000 g to collect the heavy membrane fraction. After removal of the supernatant, the remaining pellet was resuspended in isolation buffer and separated by SDS–PAGE.

### Real-time quantitative PCR

RNA was extracted using the RNeasy Mini Kit (Qiagen) and reverse transcribed to complementary DNA with the QuantiTect Reverse Transcription Kit (Qiagen) according to the manufacturer's instructions. Quantitative PCR was performed with standard TaqMan primers and TaqMan Universal Mastermix II (Applied Biosystems) on a 7500 Fast RealTime PCR system (Applied Biosystems). Fold difference was calculated by the comparative C_T_(2^−ΔΔC^T) method against Gapdh or 18 s^66^. Primers used targeted mouse Rab5a (Integrated DNA Technologies; assay ID: Mm.PT.56a.10093885), Rab5b (Integrated DNA Technologies; assay ID: Mm.PT.56a.29871709) and Rab5c (Integrated DNA Technologies; assay ID: Mm.PT.56a.28760014). Male and female WT C57BL/6 mice, age 8–12 weeks, were used for quantitative PCR of hearts. All animal protocols were in accordance with institutional guidelines and approved by the Institutional Animal Care and Use Committee of the University of California, San Diego.

### Electron microscopy

For the correlated light and electron microscopy, cells were plated on gridded MatTek dishes (MatTek Corporation) and transfected with GFP-Rab5, nanobody against GFP (VHH)-APEX2 (ref. 67), HA-Parkin and mPlum-mito-3 using Fugene 6 (Promega Inc.) according to the manufacturer's instructions. Cells were treated with 25 μM FCCP or DMSO for 4 h, then fixed in 2.5% glutaraldehyde in 0.1 M cacodylate buffer pH 7.4 for 1 h on ice and imaged on a Leica SPE II (Leica) inverted confocal microscope. For VHH-APEX2, the diaminobenzidine staining was performed as previously described^24^. Cells were postfixed in 1% osmium tetroxide for 30 min on ice. After several washes in cold double distilled water, cells were dehydrated in a cold graded ethanol series (20, 50, 70, 90 and 100%) 3 min each on ice, then washed in room temperature 100% ethanol and embedded in Durcupan ACM resin (Electron Microscopy Sciences, Hatfield, PA). Sections were cut using a diamond knife (Diatome) at a thickness between 200 and 300 nm for electron tomography and collected on LuxFilm grids (Luxel Corporation). Colloidal gold particles (5 and 10 nm diameter) were deposited on each side of the sections to serve as fiducial markers. EM data were obtained using FEI Titan high base microscope operated at 300 kV; micrographs were produced using a 4 k × 4 k Gatan charge-coupled device camera (US4000). For each section, montages of the cell of interest were acquired using the SerialEM software package. The set of two-dimensional EM maps of the cell under scrutiny was correlated with the 3D light microscopy stack allowing a global registration of the two modalities. This enabled the EM tracking of puncta representing the co-localization of the GFP-Rab5 and the mitochondrial signal in the light microscopy volume. This was accomplished using an in-house software allowing side-by-side comparison of multiple views across different microscopy modalities and establishing an optimal 3D registration. For tomography, double-tilt series were collected using the SerialEM package. For each series, the sample was tilted from −60 to +60 degrees, every 0.5 degree. Tomograms were generated using iterative reconstruction procedure^68^. To analyse larger volumes consecutive sections were reconstructed serially. The 3D reconstruction modelling was done with the IMOD tomography software package^69^.

### Statistical analysis

All experiments were independently repeated in the laboratory. Data were collected from experiments performed in at least triplicate and expressed as mean±s.e.m. No statistical method was used to predetermine sample size. Differences between groups were assayed using repeated-measure analysis of variance tests with Dunnett's *post-hoc* test, as determined by the data type. The data meet the assumptions of this test, and variances are similar between the groups that are being compared. Differences were considered to be significant when *P*<0.05. Analyses were done unblinded with respect to sample identity. Data were excluded if transfection efficiency was <40% or if cells appeared unhealthy in control conditions. Panels in Figs 1a,c,d,g and 2a,b,g, Figs 5a,d and 6a,c,f,h,j, Figs 7c,g and 8a,f, and Supplementary Figs 1a,b,c,e, 2a,d, 3a,c,e, 4a,d,g, 8a,c,e,f, 9a,c,e and 10d,f show representative images from experiments that were repeated at least three times.

### Data availability

All relevant data are available from the authors. All correlated electron microscopy image data, tomograms, segmentations and full-resolution movies can be accessed for downloading in the Cell Centered Database and Cell Image Library (http://www.cellimagelibrary.org/images?k=project_20381&simple_search=Search) under the project ID 20381.

## Additional information

**How to cite this article:** Hammerling, B. C. *et al*. A Rab5 endosomal pathway mediates Parkin-dependent mitochondrial clearance. *Nat. Commun.* **8,** 14050 doi: 10.1038/ncomms14050 (2017).

**Publisher's note:** Springer Nature remains neutral with regard to jurisdictional claims in published maps and institutional affiliations.

## Supplementary Material

Supplementary Information Supplementary Figures

Supplementary Movie 1 Electron tomography of mitochondria sequestered inside endosomes in WT MEF. Four distinct endosomes all containing a single mitochondrion can be seen (one at bottom center, three at top center) after FCCP treatment. Single slice images of these puncta are found in Supplementary Fig. 5b,d. This movie and associated data can be accessed for downloading in the Cell Centered Database and Cell Image Library (http://www.cellimagelibrary.org/images?k=project_20381&simple_search=Search) under the project ID 20381.

Supplementary Movie 2 Electron tomography of mitochondria sequestered inside endosomes in Atg5-/- MEF with 3D model overlay. Two distinct endosomes both containing a single mitochondrion can be seen (top center and bottom center) after FCCP treatment. A third endosome, adjacent to the top endosome, can also be seen. Single slice images of these puncta are found in Figure 3B (top enlarged box), and Supplementary Figure 5i. In the top left-hand corner, a mitochondrion is in the process of being engulfed by an endosome. This event has been traced and 3D-modeled in Figure 4e. 3D Modeling clearly shows spherical mitochondria contained within early endosomes. This movie and associated data can be accessed for downloading in the Cell Centered Database and Cell Image Library (http://www.cellimagelibrary.org/images?k=project_20381&simple_search=Search) under the project ID 20381.
