# Supplementary material for: A Rab5 endosomal pathway mediates Parkin-dependent mitochondrial clearance
Source: Nat Commun. 2017 Jan 30;8:14050. doi: 10.1038/ncomms14050 (PMC5290275; doi:10.1038/ncomms14050)
Supplement: Supplementary Information — Supplementary Figures [file ncomms14050-s1.pdf]

Supplementary Figure 1.

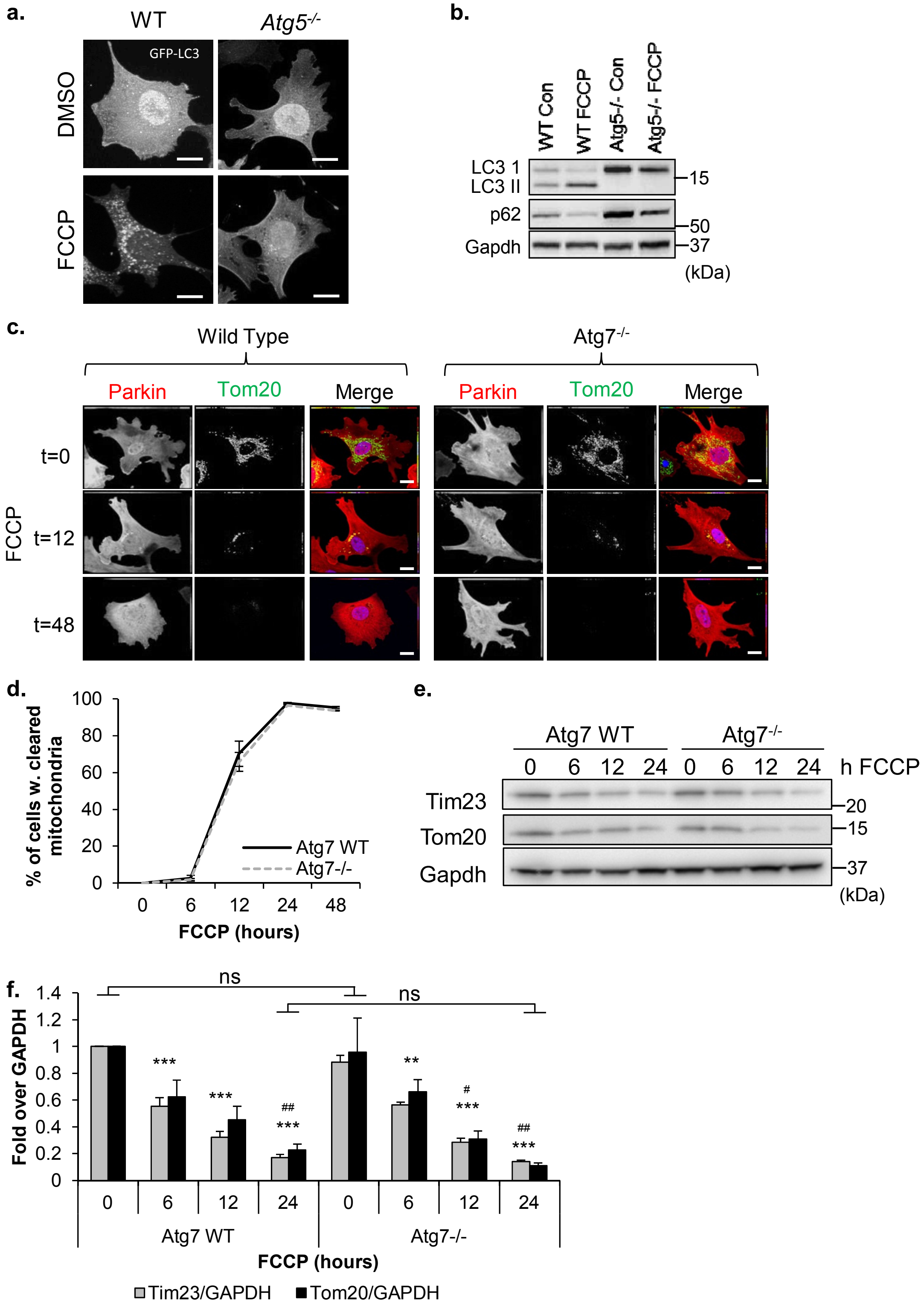

Supplementary Figure 1. Autophagy machinery is not required for mitochondrial clearance.
(**a**) Representative images of WT and *Atg5*$^{-/-}$ MEFs overexpressing GFP-LC3. Scale bars=20 μm. (**b**) Western blot for LC3, p62, and Gapdh in WT and *Atg5*$^{-/-}$ MEFs overexpressing Parkin after treatment with 25 μM FCCP for 9 h. (**c**) Representative images of primary WT and *Atg7*$^{-/-}$ MEFs infected with mCherry-Parkin and treated with DMSO or 25 μM FCCP. Cells were fixed at the indicated time points (in hours) and stained with anti-Tom20 to label mitochondria. Scale bars=20 μm. Nuclei were counterstained with Hoechst 33342 (blue). (**d**) Quantification of WT and *Atg7*$^{-/-}$ MEFs with cleared mitochondria by Tom20 staining after 25 μM FCCP treatment (n=150 cells screened for mitochondria in 3 independent experiments). (**e**) Representative western blot time course of Tim23 and Tom20 protein levels in WT and *Atg7*$^{-/-}$ MEFs overexpressing Parkin after FCCP treatment (25 μM). (**f**) Band densitometry of Tim23 and Tom20 protein levels from panel **e** (n=3, **p<0.01, ***p<0.001 vs 0 h Tim23; #p<0.05, ##p<0.01 vs 0 h Tom20). All values are means±s.e.m from independent experiments. Statistical significance was calculated using ANOVA followed by Dunnett's test for multiple comparison. Unprocessed original scans of blots are shown in Supplementary Fig. 11.

Supplementary Figure 2.

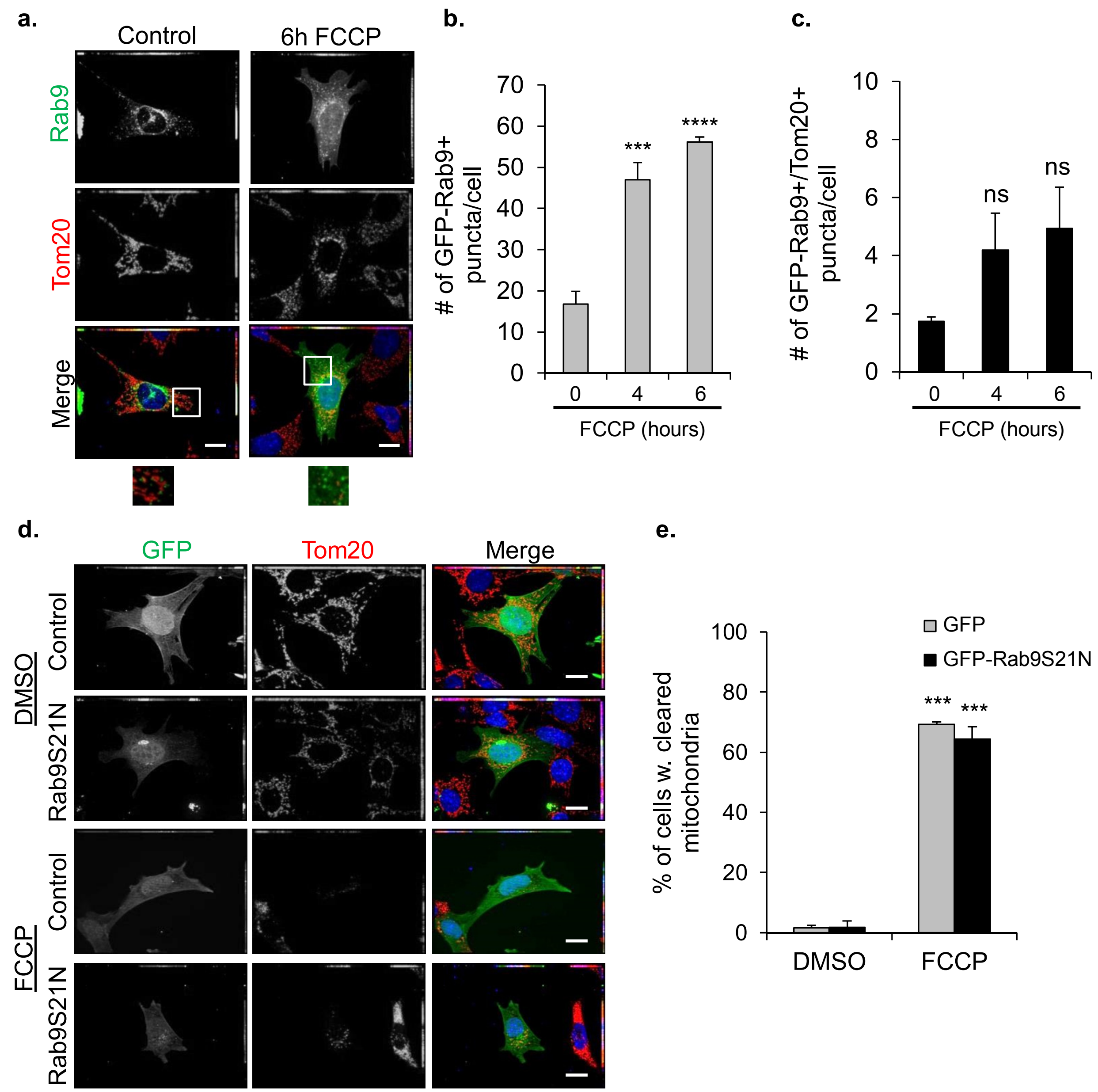

Supplementary Figure 2. Rab9-mediated alternative autophagy does not contribute to Parkin-mediated clearance in *Atg5$^{-/-}$* MEFs.

(**a**) Representative images of *Atg5$^{-/-}$* MEFs transfected with GFP-Rab9 and HA-Parkin. After 25 µM FCCP treatment, cells were fixed and stained with anti-Tom20 to label mitochondria. Scale bars=20 µm. (**b,c**) Quantification of GFP-Rab9 positive puncta (**b**) and their co-localization (**c**) with Tom20 labeled mitochondria in *Atg5$^{-/-}$* MEFs (n=45 cells scored for number of puncta in 3 independent experiments, ***p<0.001, ****P<0.0001 vs 0 h, ns=not significant). (**d**) Representative images of *Atg5$^{-/-}$* MEFs transfected with HA-Parkin plus GFP or GFP-Rab9S21N. After treatment with FCCP for 24 h, cells were fixed and stained with anti-Tom20. Scale bars=20 µm. (**d**)

Quantification of mitochondrial clearance in response to 25 μM FCCP (n=200 cells screened for mitochondria in 3 independent experiments, ***p<0.001 vs GFP+DMSO) cells. Nuclei were counterstained with Hoechst 33342 (blue). All values are means±s.e.m from independent experiments. Statistical significance was calculated using ANOVA followed by Dunnett's test for multiple comparison.

Supplementary Figure 3.

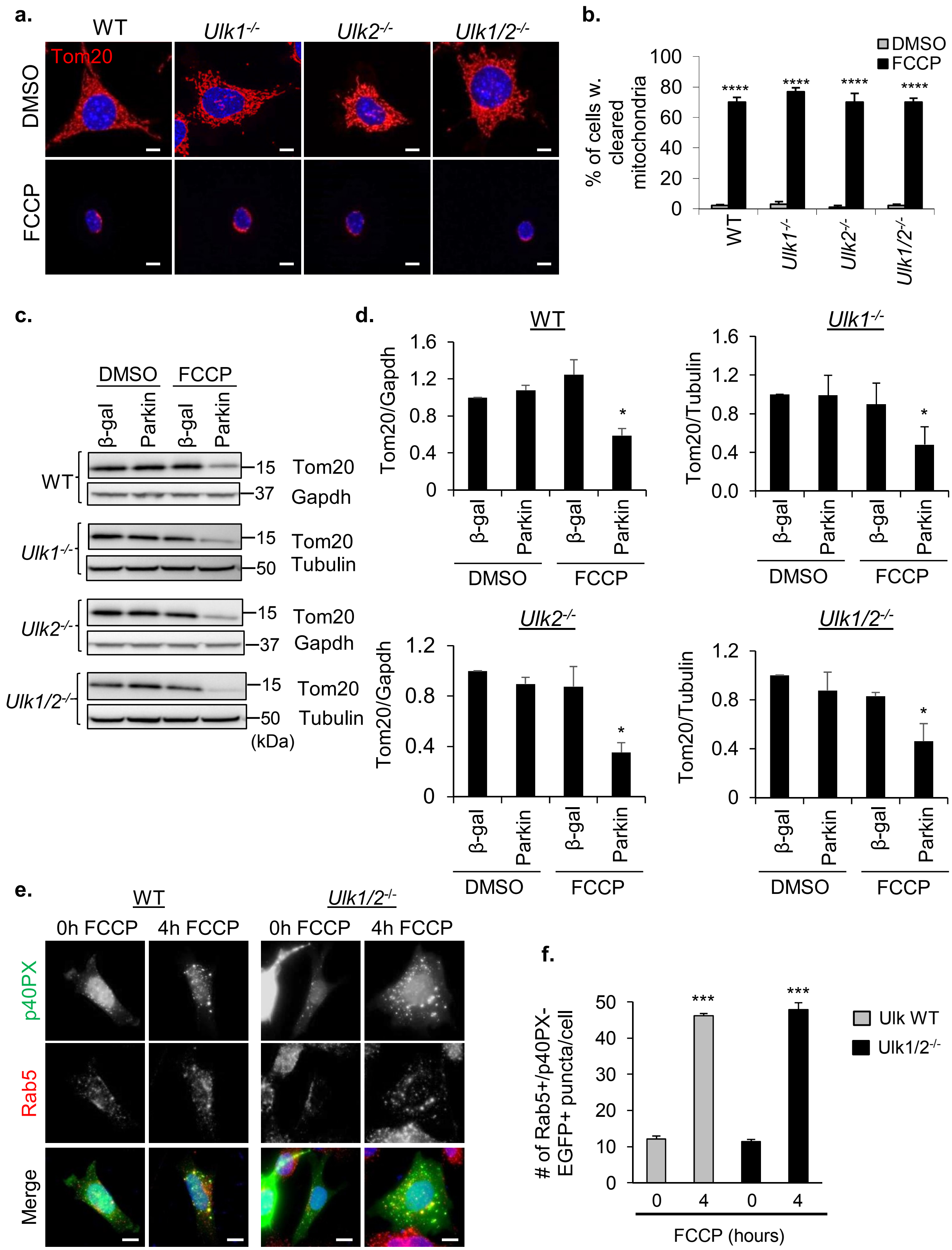

Supplementary Figure 3. Ulk1/2 are not required for Parkin-mediated clearance of damaged mitochondria.
(**a**) Representative images of WT, *Ulk1*$^{-/-}$, *Ulk2*$^{-/-}$, and *Ulk1/2*$^{-/-}$ MEFs transfected with HA-Parkin, treated with 25 µM FCCP (24 h), and stained with anti-Tom20 to label mitochondria. Scale bars=20 µm. (**b**) Quantification of cells undergoing mitochondrial clearance (n=550 cells screened for mitochondria in 3 independent experiments, ****p<0.0001 vs DMSO). (**c**) Representative Western blots for Tom20, Tubulin, and GAPDH in WT, *Ulk1*$^{-/-}$, *Ulk2*$^{-/-}$, and *Ulk1/2*$^{-/-}$ MEFs infected with Ad-βgal or Ad-Parkin after treatment with DMSO or FCCP (25 µm) for 24 h. (**d**) Band densitometry of Tom20 levels (n=3, *p<0.05 vs β-gal + FCCP). (**e**) Representative images of WT or *Ulk1/2*$^{-/-}$ transfected with p40PX-EGFP and treated with 25 µM FCCP for 0 or 4 h. After treatment, cells were fixed and stained with anti-Rab5. Scale bars=20 µm. (**f**) Quantification of colocalization between Rab5 and p40PX-EGFP positive puncta (n=30 cells scored for number of puncta in 3 independent experiments, ***p<0.001). Nuclei were counterstained with Hoechst 33342 (blue). All values are means±s.e.m from independent experiments. Statistical significance was calculated using ANOVA followed by Dunnett's test for multiple comparison. Unprocessed original scans of blots are shown in Supplementary Fig. 11.

Supplementary Figure 4.

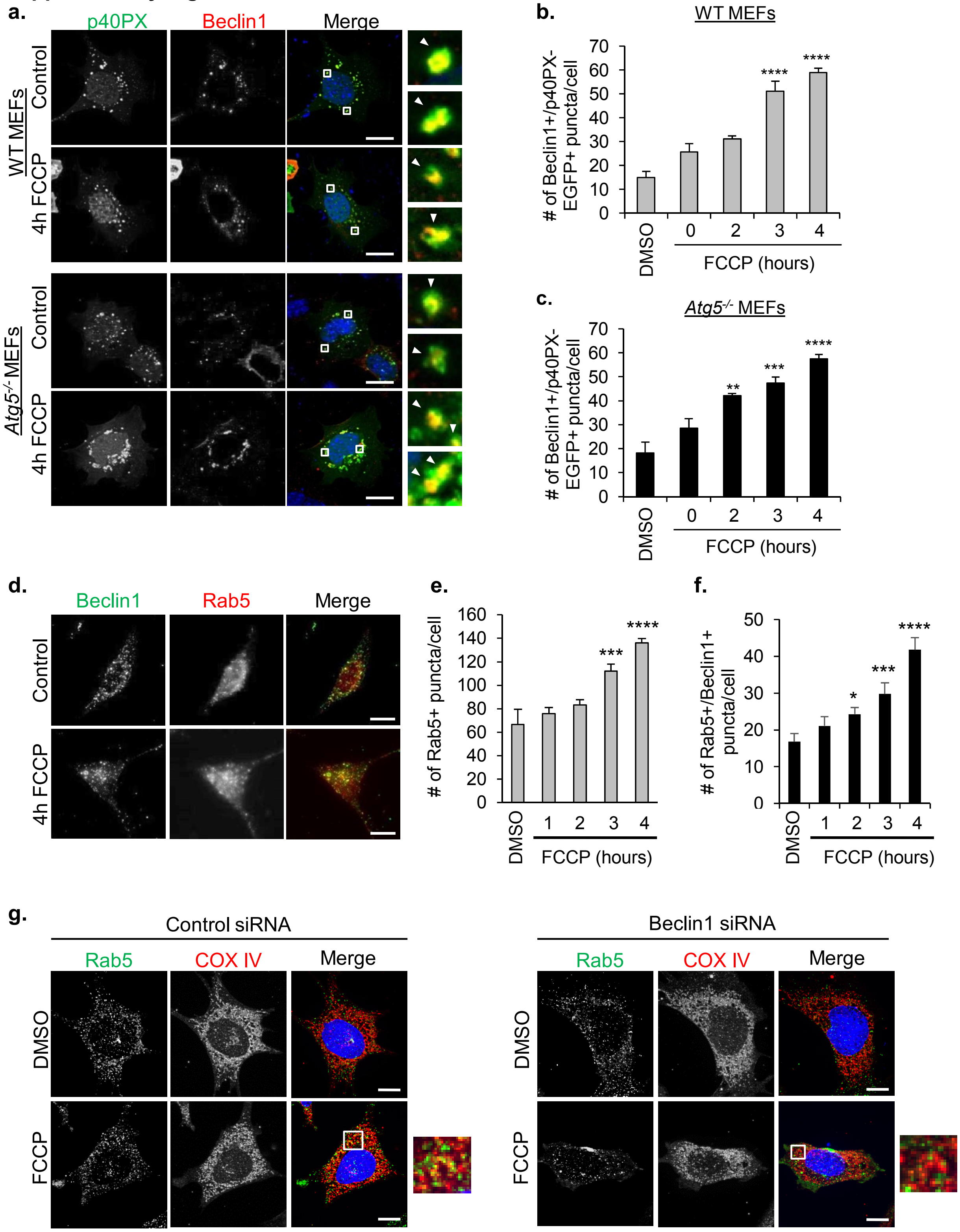

Supplementary Figure 4. **Beclin1-associated Class III PI3K activity is increased in WT and *Atg5*$^{-/-}$ MEFs in response to FCCP treatment.**

(**a**) Representative images of WT and *Atg5*$^{-/-}$ MEFs overexpressing p40PX-EGFP, and HA-Beclin1. Cells were treated with DMSO or FCCP (25 μM) for 4 h. Arrowheads show colocalizing puncta. Scale bars=20 μm. (**b,c**) Quantification of puncta positive for p40PX-EGFP and HA-Beclin1 in WT (**b**) and *Atg5*$^{-/-}$ (**c**) cells (n=40 cells scored for number of puncta in 4 independent experiments, **p<0.01, ***p<0.001, ****p<0.0001 vs 0 h). (**d**) Representative images for *Atg5*$^{-/-}$ MEFs expressing HA-Beclin1, treated with DMSO or 25 μM FCCP and stained with anti-HA and anti-Rab5. Scale bars=20 μm. (**e,f**) Quantification of Rab5 positive puncta (**e**) and their co-localization (**f**) with Beclin1 in response to treatment (n=35 cells scored for number of puncta in 4 independent experiments, *p<0.05, ***p<0.001, ****p<0.0001 vs DMSO). (**g**) Representative images of Parkin-expressing *Atg5*$^{-/-}$ MEFs transfected with control or Beclin1 siRNA, treated with DMSO or FCCP (25 μM) for 4 h. Fixed cells were stained with anti-Rab5 and anti-COX IV. Scale bars=20 μm. Nuclei were counterstained with Hoechst 33342 (blue). All values are means±s.e.m from independent experiments. Statistical significance was calculated using ANOVA followed by Dunnett's test for multiple comparison.

Supplementary Figure 5.

WT MEFs

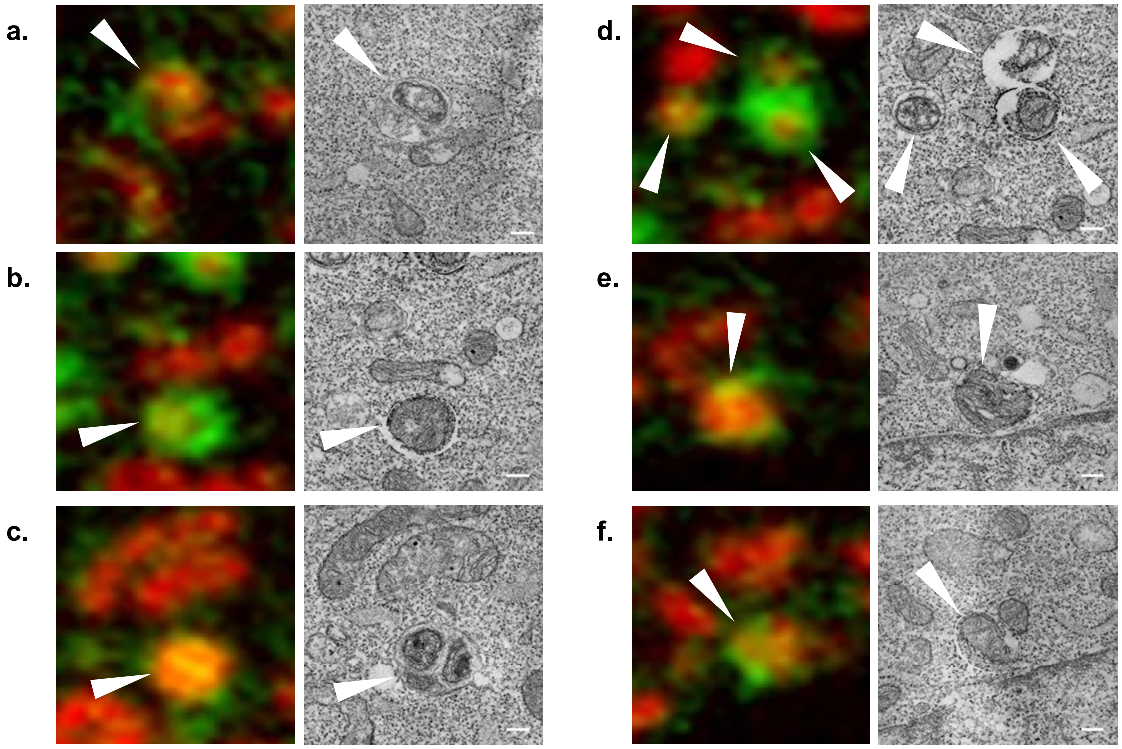

Atg5-/- MEFs

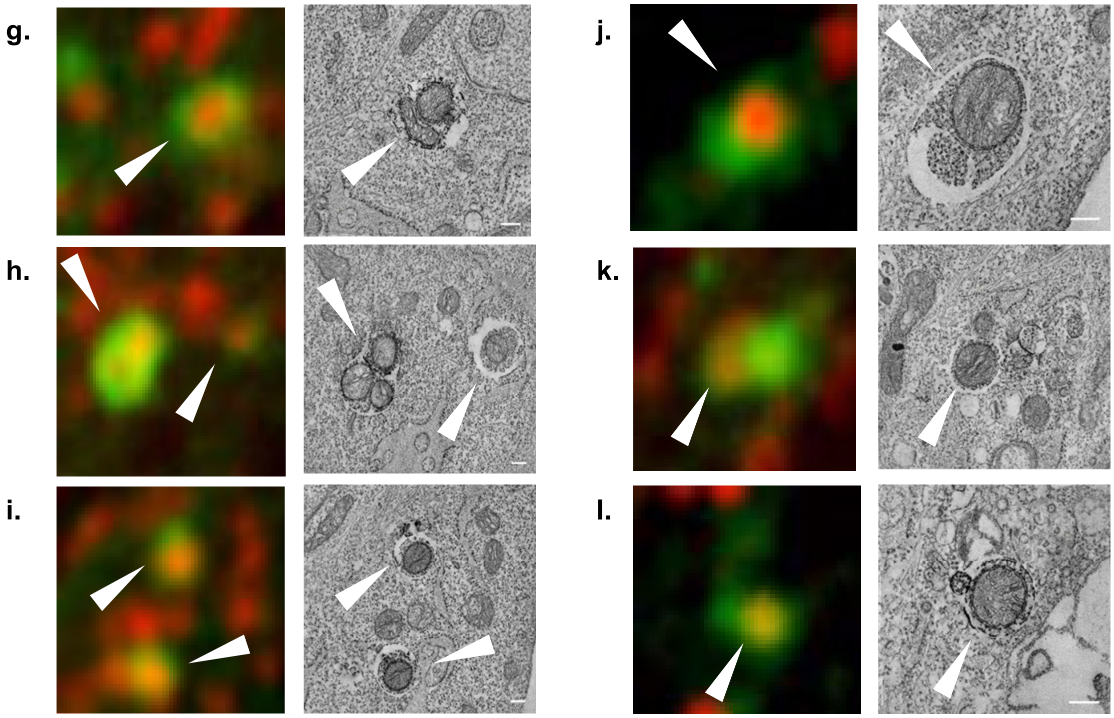

Supplementary Figure 5. Mitochondria are found inside GFP-Rab5 positive endosomes in WT and *Atg5*$^{-/-}$ MEFs after FCCP treatment.

Arrowheads mark instances of co-localization between mPlum-mito tagged mitochondria (red) and GFP-Rab5 endosomes (green) in WT (**a-f**) and *Atg5*$^{-/-}$ (**g-l**) MEFs after FCCP (25 μM, 4 h) treatment (colored images). Related to Figure **3a,b**. Correlated electron microscopy of these puncta show mitochondria inside the lumen of single-membrane vesicles. Panels **h** and **i** represent the same puncta from Figure 3B. Tomograms of puncta in panels **d** and **i** can also be found in Supplementary Movies 1 and 2, respectively. Scale bars= 200 nm.

Supplementary Figure 6.

a.

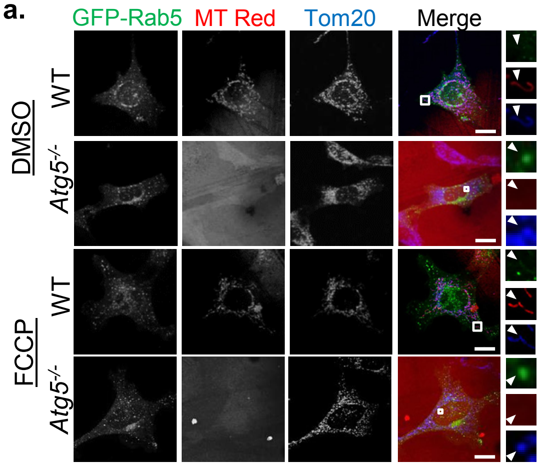

b.

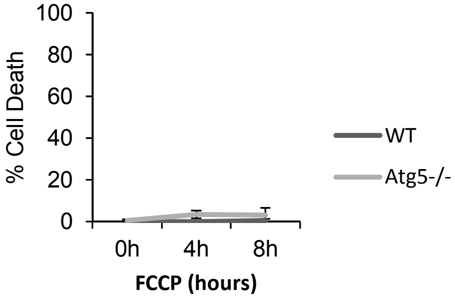

Supplementary Figure 6. Depolarized mitochondria are sequestered by Rab5 positive endosomes in the absence of cell death.

(**a**) Representative images of WT and *Atg5*$^{-/-}$ MEFs transfected with GFP-Rab5 and HA-Parkin, treated with DMSO or 25 µM FCCP for 4 h, and stained with MitoTracker Red CMXRos (MT Red). After treatment, cells were fixed and stained with anti-Tom20. Scale bars=20 µm. Arrowheads show colocalizing puncta. (**b**) Quantification of cell death. WT or *Atg5*$^{-/-}$ cells overexpressing mCherry-Parkin were exposed to FCCP (25 µM) for 0, 4, or 8 h and stained with Yo-Pro-1 (n=100 cells screened for cell death in 3 independent experiments).

Supplementary Figure 7.

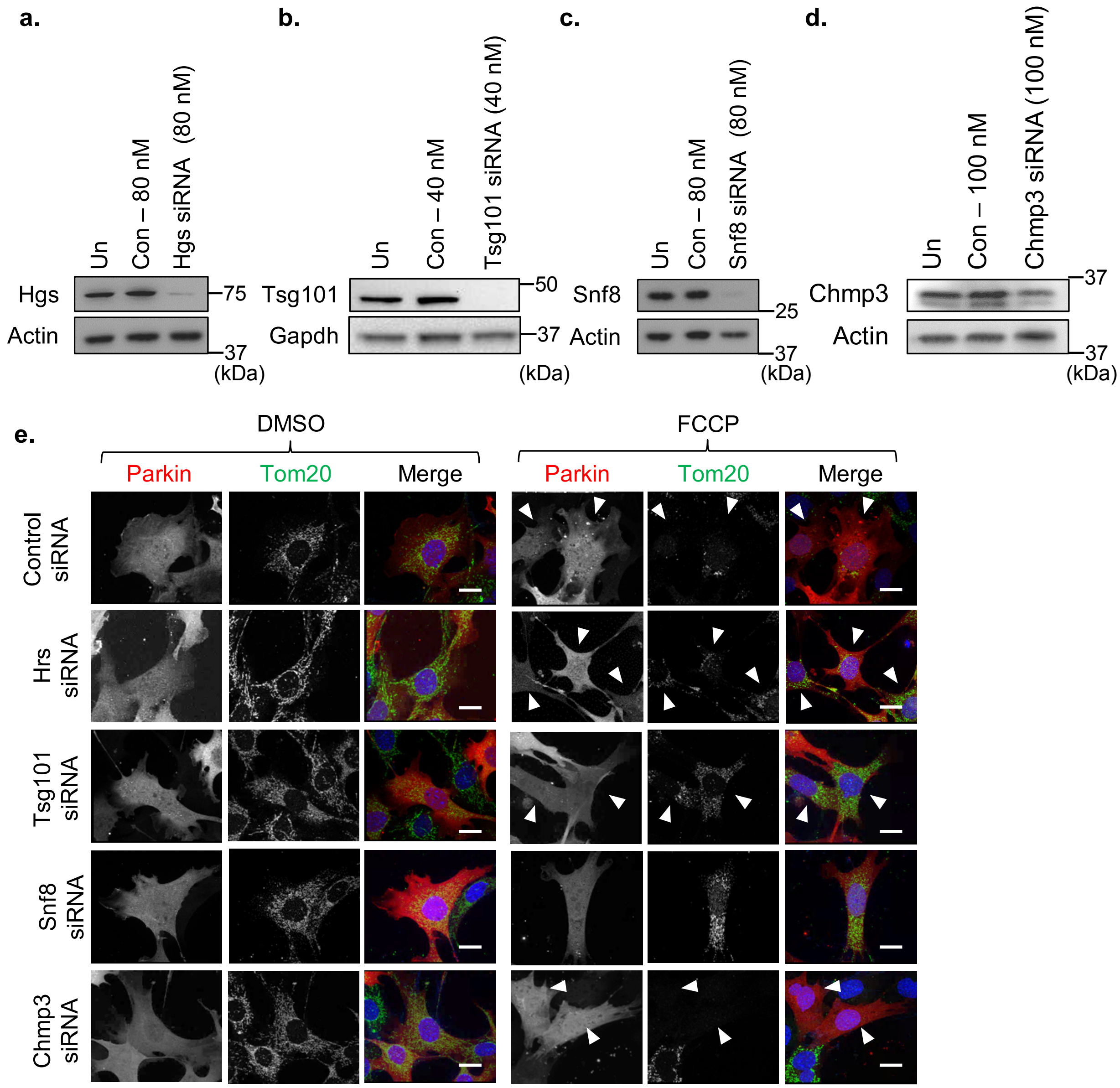

Supplementary Figure 7. Confirmation of ESCRT knockdown and the effect on mitochondria clearance.

(**a-d**) Western blots confirming siRNA knockdown of Hgs (**a**), Tsg101 (**b**), Snf8 (**c**), and Chmp3 (**d**) by siRNA after 96h. Un= untransfected, con= control siRNA transfected. (**e**) Images showing mitochondrial clearance after ESCRT protein knockdown. After ESCRT protein knockdown, *Atg5*$^{-/-}$ cells were infected with mCherry-Parkin, and treated with DMSO or 25 μM FCCP for 12 h. Fixed cells were stained with anti-Tom20 to label mitochondria. Nuclei were counterstained with Hoechst 33342 (blue). Arrowheads indicate individual cells. Scale bars=20 μm. Unprocessed original scans of blots are shown in Supplementary Fig. 11.

Supplementary Figure 8.

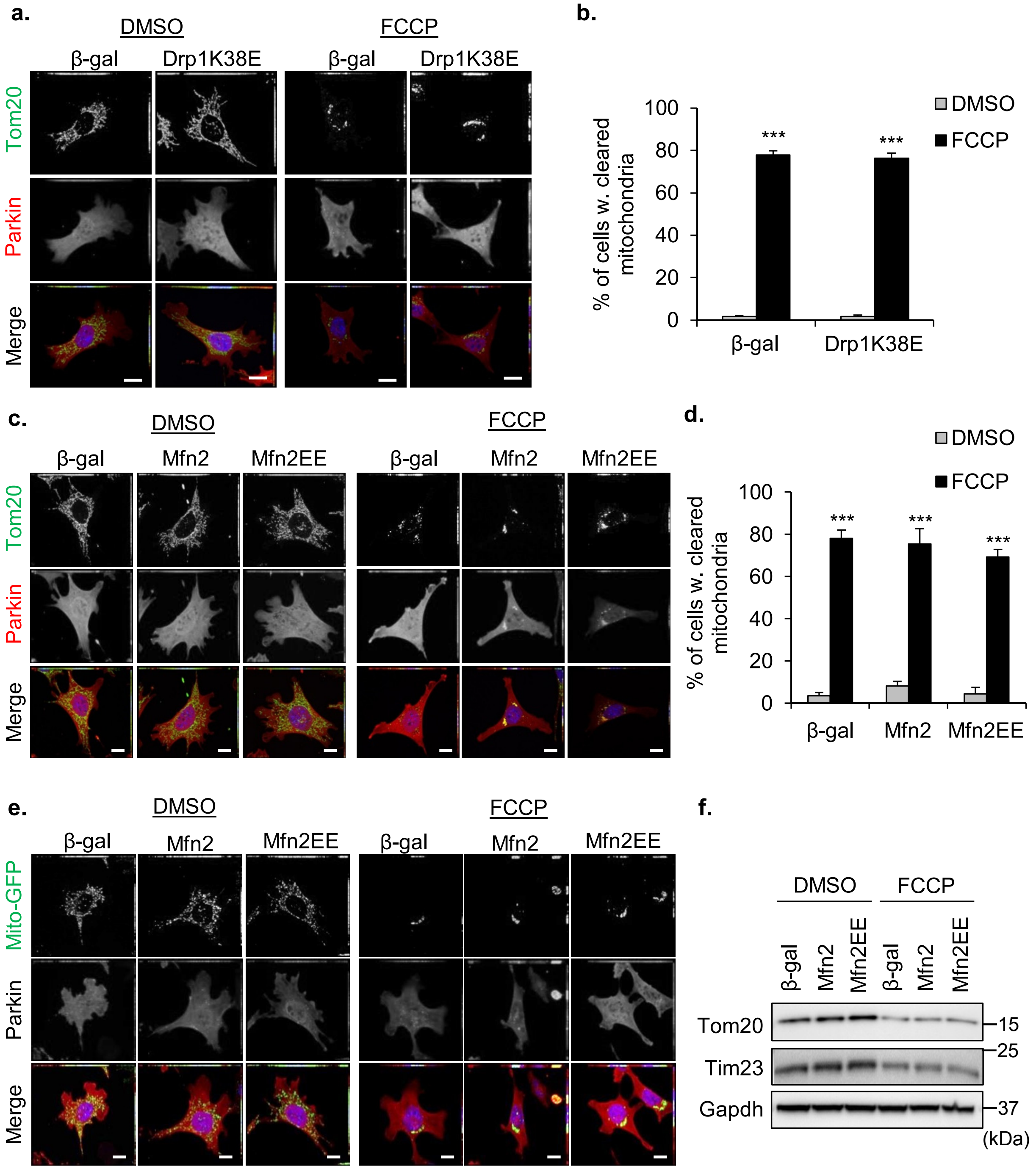

Supplementary Figure 8. Mitochondrial fission is not required for Parkin-mediated clearance in *Atg5$^{-/-}$* MEFs.

(**a**) Representative images of *Atg5$^{-/-}$* MEFs overexpressing Parkin and β-gal or Drp1K38E. After 25 μM FCCP treatment (24 h), cells were fixed and stained with anti-Tom20. Scale bars=20 μm. (**b**) Quantification of cells undergoing mitochondria clearance (n=200 cells screened for mitochondria in 3 independent experiments,

***p<0.001 vs DMSO). (**c**) Representative images of *Atg5*$^{-/-}$ MEFs overexpressing mCherry-Parkin plus βgal, Mfn2 or Mfn2EE. After treatment with 25 μM FCCP for 24 h, cells were fixed and stained with anti-Tom20. Scale bars=20 μm. (**d**) Quantification of cells undergoing mitochondria clearance (n=150 cells screened for mitochondria in 3 independent experiments, ***p<0.001 vs DMSO). (**e**) Representative images of *Atg5*$^{-/-}$ MEFs overexpressing mCherry-Parkin and Mito-GFP (localizes to mitochondrial matrix) plus Mfn2 or Mfn2EE. Scale bars=20 μm. (**f**) Western blot for Tom20, Tim23, and GAPDH in *Atg5*$^{-/-}$ MEFs overexpressing Parkin plus Ad-β-gal, Ad-Mfn2, or Ad-Mfn2EE. Nuclei were counterstained with Hoechst 33342 (blue). All values are means±s.e.m from independent experiments. Statistical significance was calculated using ANOVA followed by Dunnett's test for multiple comparison. Unprocessed original scans of blots are shown in Supplementary Fig. 11.

Supplementary Figure 9.

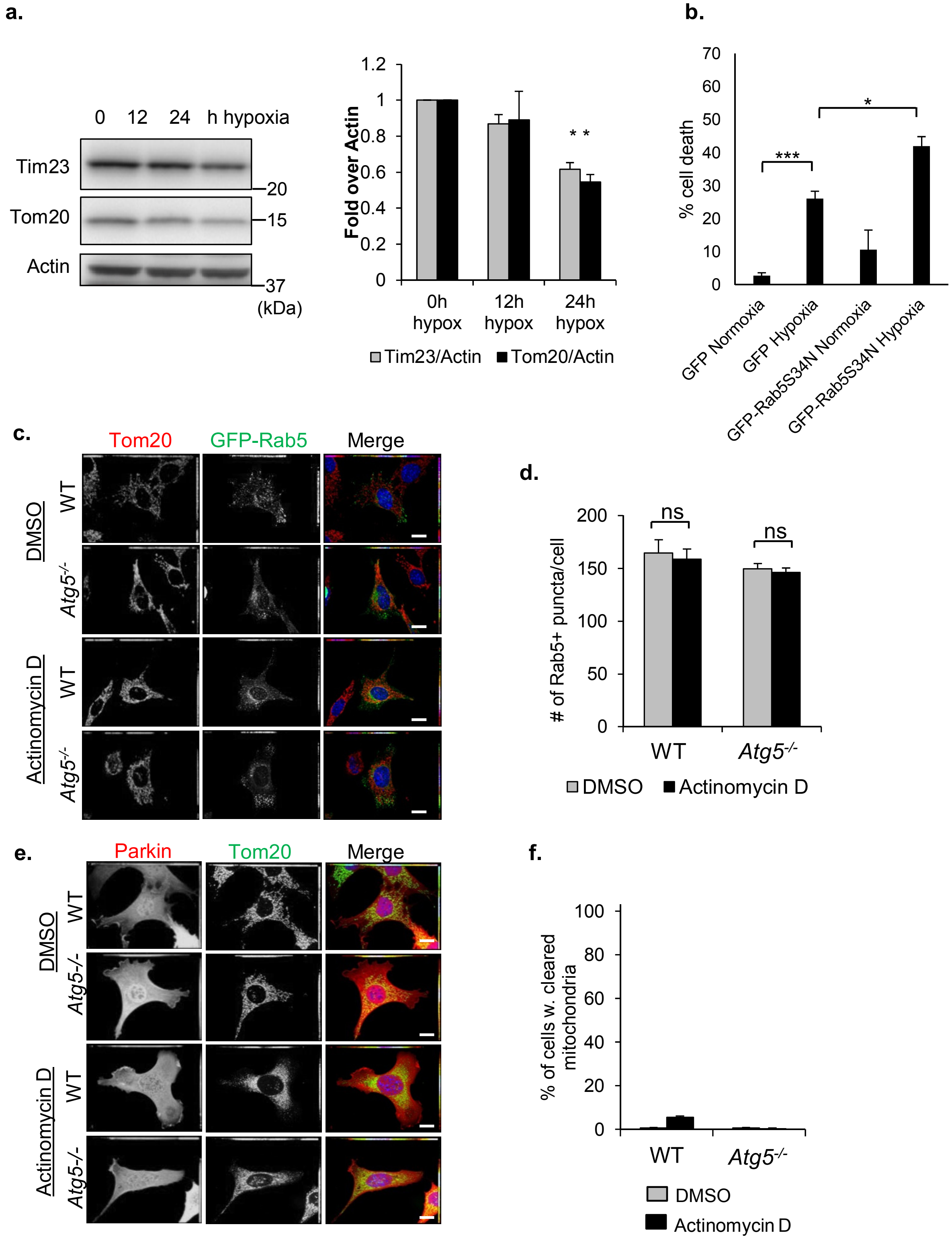

Supplementary Figure 9. Hypoxia-associated, but not DNA, damage induces mitochondrial clearance.

(**a**) Western blot for Tim23 and Tom20 in *Atg5*$^{-/-}$ MEFs overexpressing mCherry-Parkin under hypoxic conditions for the indicated length of time (left). Band densitometry of protein levels (right; n=3, *p<0.05, vs 0 h). (**b**) Quantification of cell death. *Atg5*$^{-/-}$ MEFs overexpressing myc-Parkin and GFP or GFP-Rab5S34N were exposed to normoxic or hypoxic conditions for 34 h and then stained with Po-Pro-3. (n= 200 screened for cell death in 3 independent experiments, *p<0.05, ***p<0.001). (**c**) Representative images of WT and *Atg5*$^{-/-}$ MEFs transfected with GFP-Rab5 and HA-Parkin and treated with DMSO or 0.05 µg/mL actinomycin D for 4 h. After treatment, cells were fixed and stained with anti-Tom20. Scale bars=20 µm. (**d**) Quantification of Rab5-positive vesicles (**i**, n=30 cells scored for number of puncta in 3 independent experiments, ns=not significant). (**e**) Representative images of WT and *Atg5*$^{-/-}$ MEFs overexpressing mCherry-Parkin and treated with DMSO or actinomycin D (0.05 µg/mL) for 24 h. Cells were stained with anti-Tom20. (**f**) Quantification of mitochondrial clearance (n=200 cells screened for mitochondria in 3 independent experiments) in response to actinomycin D. Scale bars=20 µm. Nuclei were counterstained with Hoechst 33342 (blue). All values are means±s.e.m from independent experiments. Statistical significance was calculated using ANOVA followed by Dunnett's test for multiple comparison. Unprocessed original scans of blots are shown in Supplementary Fig. 11.

Supplementary Figure 10.

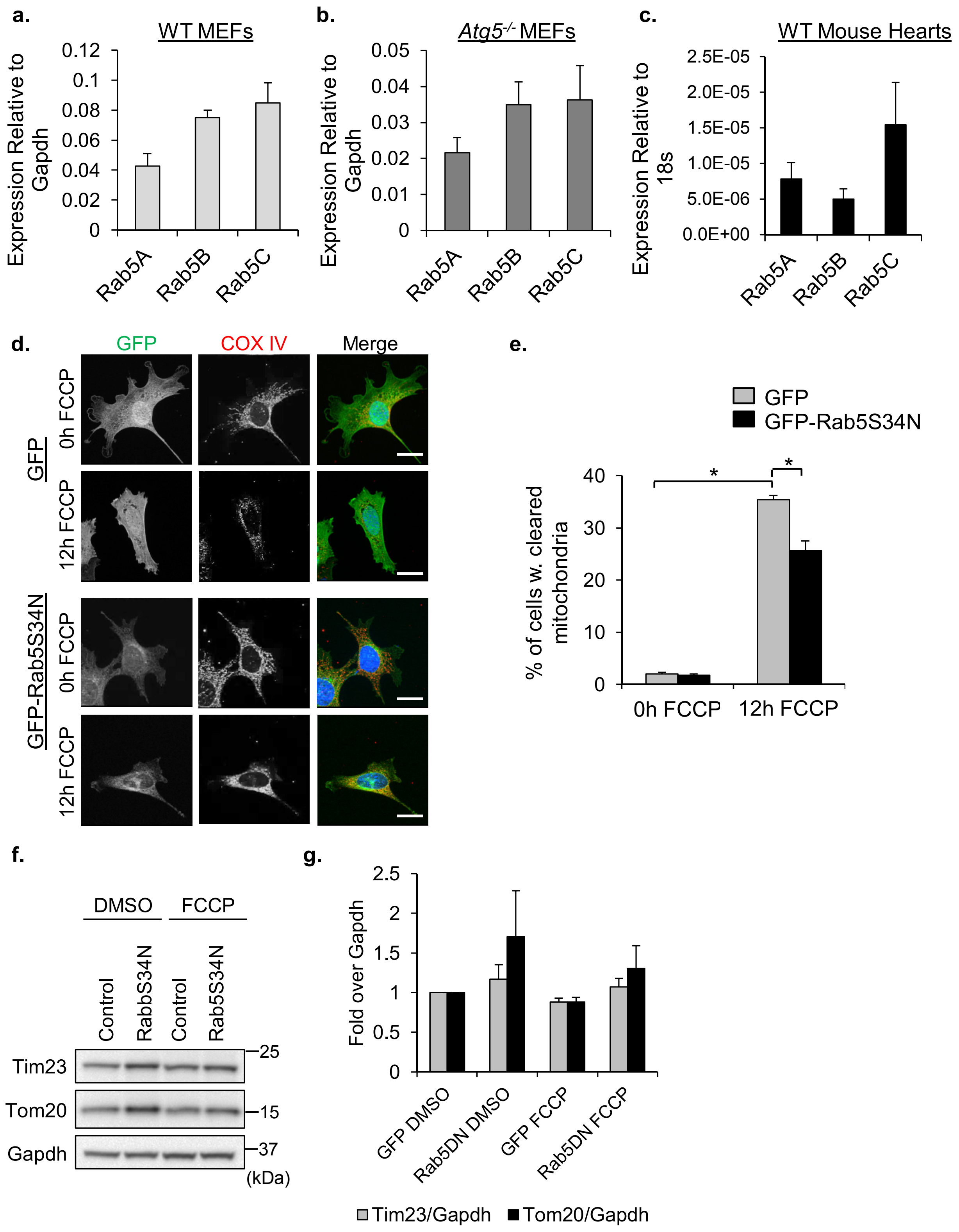

Supplementary Figure 10. Inhibition of Rab5 impairs mitochondrial clearance. (**a,b,c**) qPCR for Rab5 isoforms in WT MEFs (**a**), *Atg5*$^{-/-}$ MEFs (**b**), and mouse hearts (**c**). Data from 3 independent experiments (**a, b**), or from 5 hearts (**c**). (**d**) Representative images of *Atg5*$^{-/-}$ MEFs expressing Parkin infected with GFP or GFP-Rab5S34N, treated with 25 μM FCCP (0 or 12 h), and stained with anti-COX IV and Hoechst 33342 (blue). Scale bars=20 μm. (**e**) Quantification of cells undergoing mitochondria clearance (n=85 cells screen for mitochondria in 3 independent experiments, *p<0.05). (**f**) Western blot for Tim23 and Tom20 protein levels in WT MEFs expressing Parkin infected with β-gal (control) or Rab5S34N and treated with DMSO or FCCP (25 μM) for 4 h. (**g**) Band densitometry of Tim23 and Tom20 proteins levels from panel **f** (n=3, data are not significant). All values are means±s.e.m from independent experiments. Statistical significance was calculated using ANOVA followed by Dunnett's test for multiple comparison. Unprocessed original scans of blots are shown in Supplementary Fig. 11.

Supplementary Figure 11.

Figure 1c

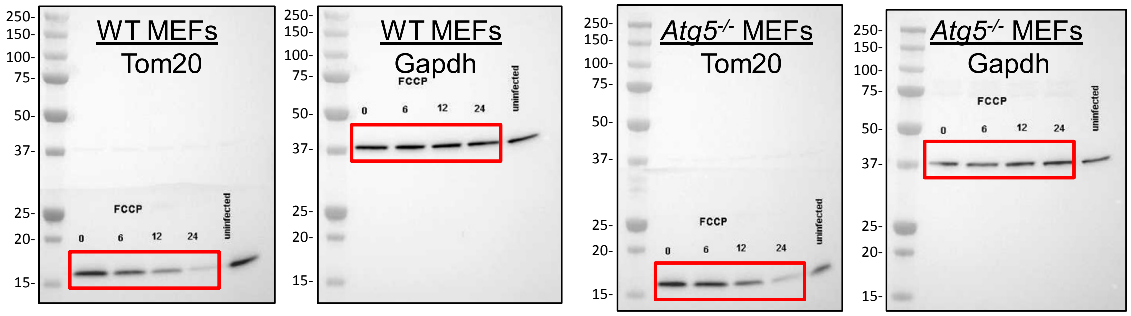

Figure 1d

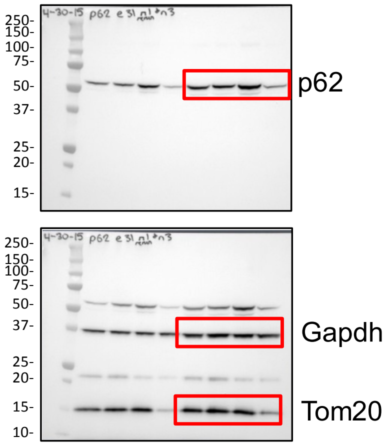

Figure 2j

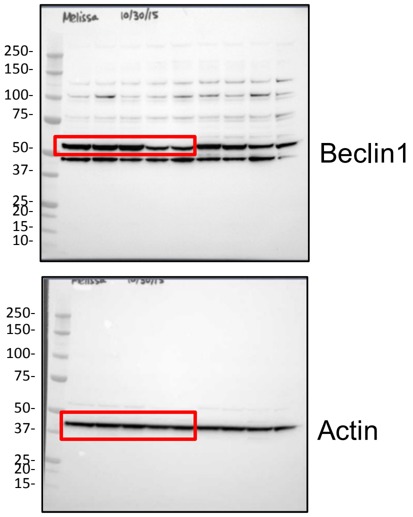

Figure 4b

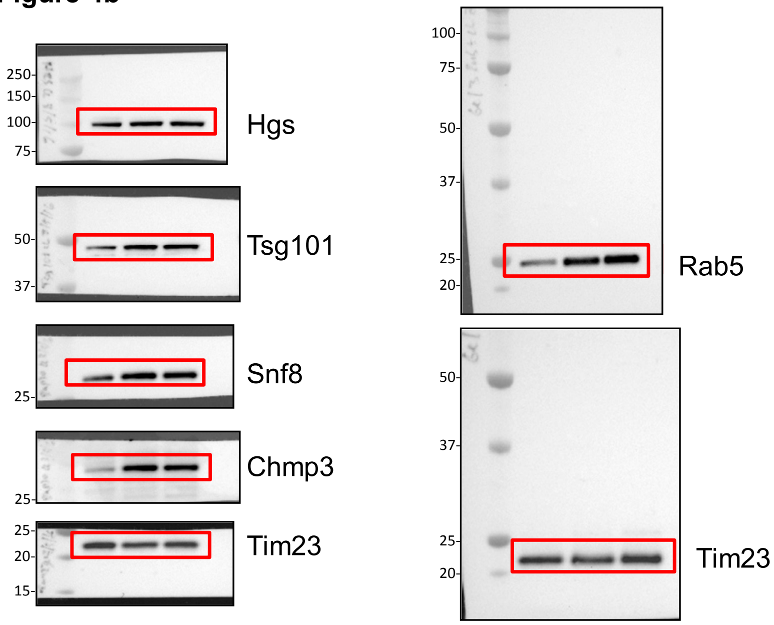

Figure 7c

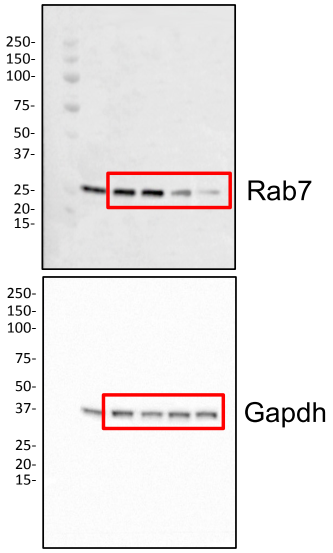

Figure 7g

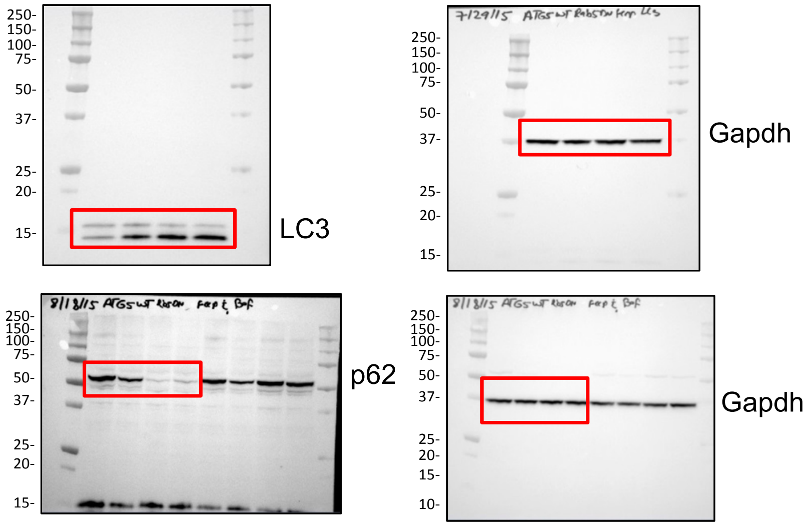

Figure 8e

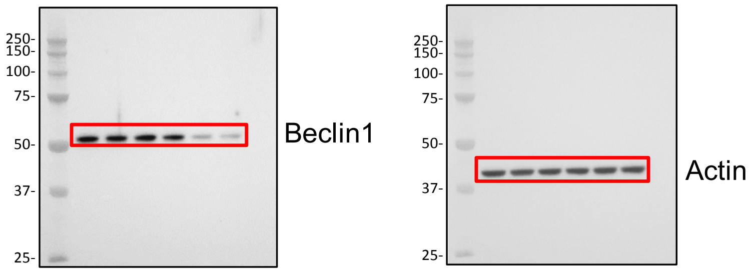

Figure S1B

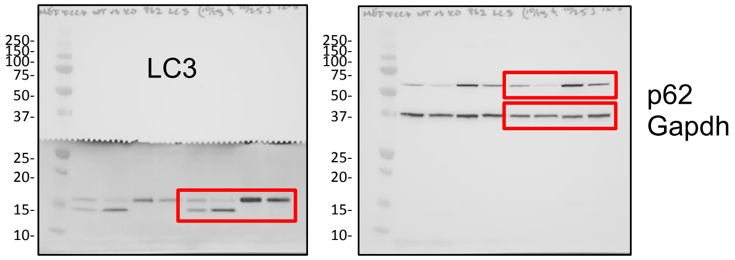

Figure S1E

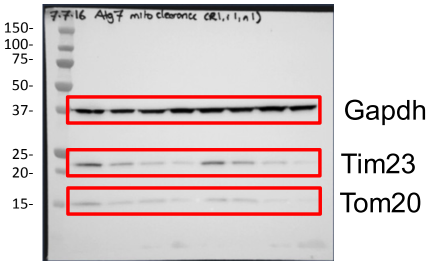

Figure S3C

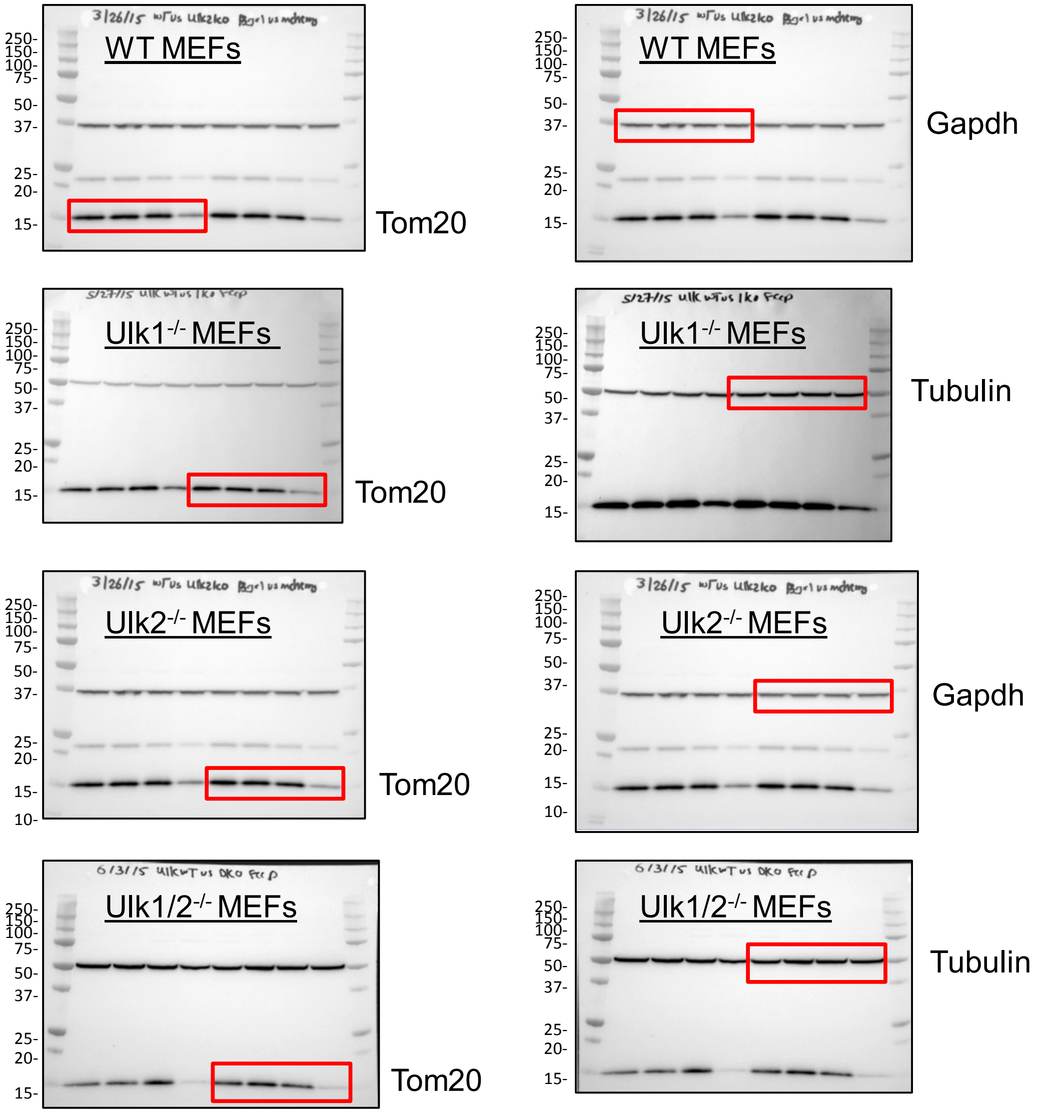

Figure S7a

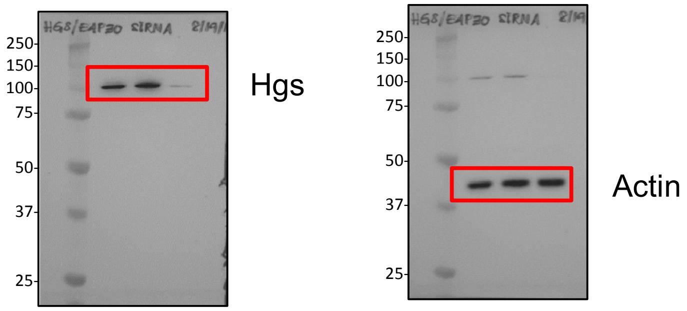

Figure S7b

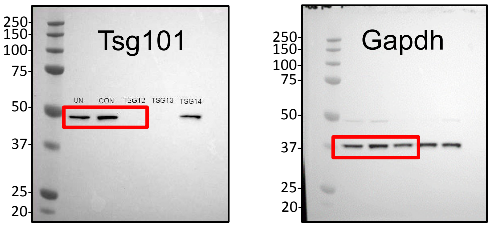

Figure S7c

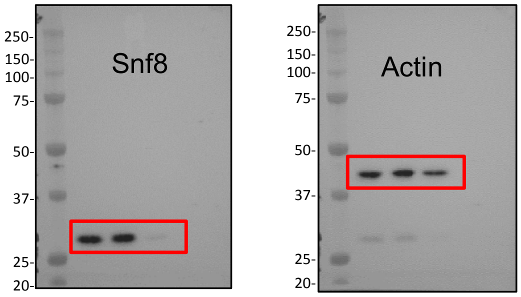

Figure S7d

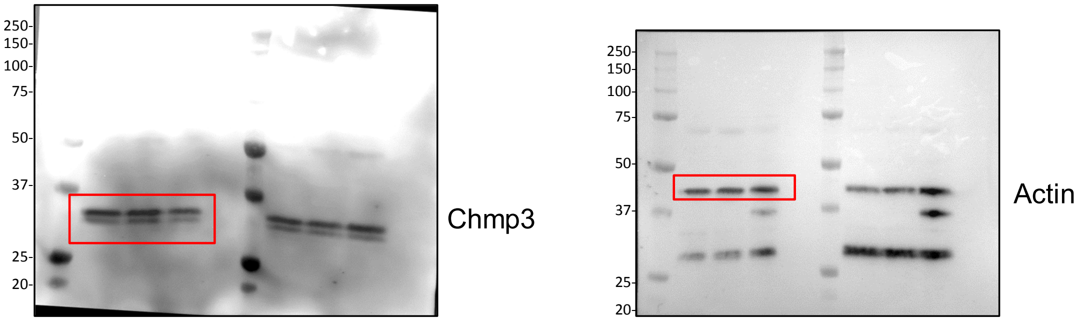

Figure S8f

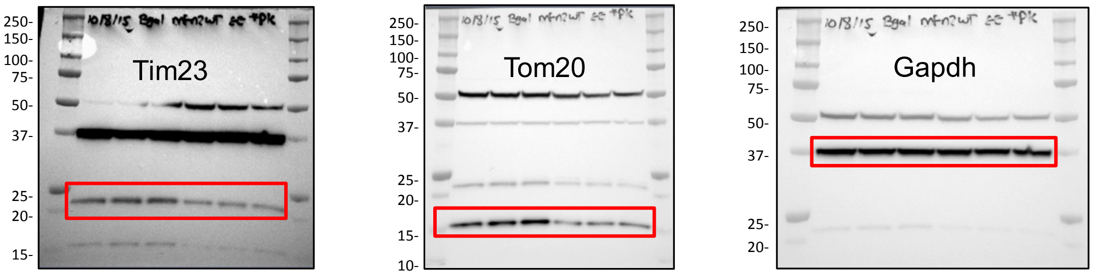

Figure S9a

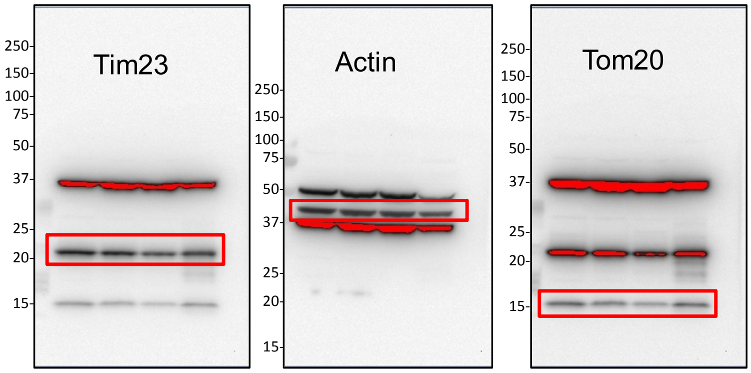

Figure S10f

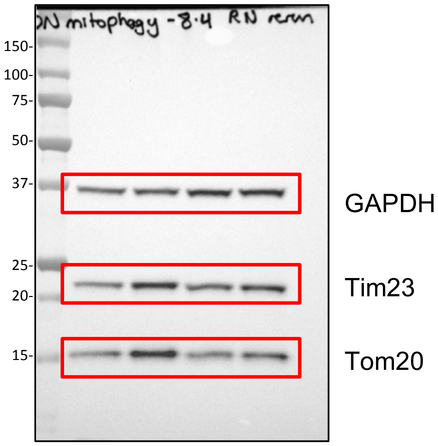

Supplementary Figure 11. Uncropped Western blots from previous figures.
